# Elevated Red Blood Cell Distribution Width Predicts Mortality and Major Adverse Cardiovascular Events After Acute Myocardial Infarction: A Large Propensity Score-Matched Cohort Study

**DOI:** 10.3390/jcm15062432

**Published:** 2026-03-22

**Authors:** Kuan-Chung Ting, Chi-Jiang Liao, Chun Lee, Ming-Jen Tsai

**Affiliations:** 1 Department of Emergency Medicine, Ditmanson Medical Foundation Chia-Yi Christian Hospital, Chiayi City 600, Taiwan; b04401027@ntu.edu.tw (K.-C.T.); matthewstliao@yahoo.com.tw (C.-J.L.); 2 Clinical Data Center, Ditmanson Medical Foundation Chia-Yi Christian Hospital, Chiayi City 600, Taiwan; cych15903@gmail.com

**Keywords:** red blood cell distribution width, myocardial infarction, mortality, major adverse cardiovascular events, prognosis

## Abstract

**Background**: Red blood cell distribution width (RDW) is an accessible prognostic biomarker in cardiovascular disease, but its independent association with clinical outcomes in patients with acute myocardial infarction (AMI) undergoing percutaneous coronary intervention (PCI) remains incompletely characterized, particularly regarding its prognostic value independent of anemia status. **Methods**: Using the TriNetX US Collaborative Network (70 healthcare organizations; >105 million patients), we identified 84,811 adult AMI patients who underwent PCI between January 2019 and December 2023 and had RDW measured on the index date. Patients were stratified by RDW ≥ 13.5% (high) versus <13.5% (low) and matched 1:1 using propensity scores based on 38 baseline characteristics. The primary outcome was 1-year all-cause mortality, assessed using a 30-day landmark approach. Secondary outcomes included major adverse cardiovascular events (MACE), heart failure, cardiogenic shock, recurrent AMI, cerebrovascular accident, ventricular tachycardia/fibrillation, and cardiac arrhythmia. **Results**: After matching (32,010 pairs), high RDW was significantly associated with increased 1-year all-cause mortality (HR 1.77, 95% CI 1.62–1.93, *p* < 0.001). High RDW was also associated with greater risks of MACE (HR 1.12), heart failure (HR 1.24), cardiogenic shock (HR 1.26), recurrent AMI (HR 1.11), cerebrovascular accident (HR 1.16), and cardiac arrhythmia (HR 1.14; all *p* < 0.01). Findings remained consistent across serial sensitivity analyses and subgroup analyses. Among non-anemic patients, high RDW remained strongly associated with mortality (HR 1.67, 95% CI 1.50–1.85, *p* < 0.001). **Conclusions**: Elevated RDW at the time of AMI is independently associated with mortality and adverse cardiovascular outcomes after PCI, including among non-anemic patients. RDW may serve as a readily available tool to support early risk stratification in this population.

## 1. Introduction

Acute myocardial infarction (AMI) remains a leading cause of morbidity and mortality worldwide despite substantial advances in reperfusion strategies and contemporary medical therapy [1]. Although percutaneous coronary intervention (PCI) has significantly improved short-term outcomes, the long-term prognosis of patients surviving the acute phase remains highly variable, underscoring the need for early and accurate risk stratification [2,3,4]. Identification of readily available biomarkers that can enhance prognostic assessment beyond traditional risk scores is therefore of considerable clinical importance [5,6].

Red blood cell distribution width (RDW), a quantitative measure of red blood cell size variability, is automatically reported as part of the standard complete blood count [7,8]. Emerging evidence suggests that RDW reflects not only hematologic abnormalities but also systemic inflammation, oxidative stress, and impaired erythropoiesis—pathophysiological processes that contribute to endothelial dysfunction and atherosclerotic plaque instability [8,9,10]. Multiple studies have demonstrated associations between elevated RDW and adverse outcomes in cardiovascular diseases, including heart failure, atrial fibrillation, acute coronary syndromes, and stroke [7,10,11]. Specifically in patients with AMI, elevated RDW has been associated with increased mortality and major adverse cardiovascular events (MACE) [8,10,12,13,14].

However, several important knowledge gaps remain. First, most prior studies were limited by small to moderate sample sizes (typically < 5000 patients), single-center designs, or short follow-up durations [8,10,12,13]. Second, given that patients with elevated RDW often have greater comorbidity burden [15], unmeasured confounding may inflate observed associations. Few studies have employed rigorous methodological approaches such as propensity score matching (PSM) to minimize confounding by baseline characteristics [16,17]. Third, it remains unclear whether the prognostic value of RDW is independent of anemia, a common condition in patients with AMI (prevalence 10–43%) that shares well-recognized pathophysiological mechanisms with elevated RDW and is itself a predictor of adverse outcomes [18,19]. Finally, the longer-term prognostic value of RDW in AMI patients has not been well established. The central unresolved question of whether elevated RDW carries independent prognostic value beyond its association with anemia in AMI patients undergoing PCI has not been addressed in any prior large-scale, rigorously controlled cohort study.

Therefore, we conducted this large-scale, multicenter retrospective cohort study using the TriNetX global health research network to investigate the association between RDW and clinical outcomes in patients with AMI who underwent PCI. We employed 1:1 PSM to minimize confounding by baseline characteristics and performed prespecified sensitivity analyses, including extended 2-year follow-up, multivariable Cox regression, and stratification by baseline hemoglobin status and AMI type, to assess the independent and robust prognostic value of RDW in this population.

## 2. Methods

### 2.1. Study Design and Data Source

This retrospective cohort study utilized data from TriNetX, a global federated health research network providing access to de-identified electronic medical records across healthcare organizations (HCOs) [20]. All personally identifiable information is removed prior to data integration, and TriNetX employs comprehensive data security measures, including encryption, access logging, and compliance with HIPAA and GDPR standards. Researchers access the platform exclusively through secure web-based interfaces, and all outputs are provided in aggregate form to minimize re-identification risk. We queried the US Collaborative Network, comprising 70 HCOs across the United States with over 105 million patients, with the final query performed on 13 March 2026. The study was approved by the Institutional Review Board of Ditmanson Medical Foundation Chiayi Christian Hospital (IRB 2026006) with a waiver of informed consent for analysis of de-identified data and followed the Strengthening the Reporting of Observational Studies in Epidemiology (STROBE) guidelines.

### 2.2. Study Population

We identified adult patients (aged ≥ 18 years) diagnosed with AMI (ICD-10-CM code I21) between 1 January 2019, and 31 December 2023, who underwent PCI within ±3 days of the AMI diagnosis. This narrow time window was selected to capture acute revascularization while accounting for inter-facility transfers and minimizing immortal time bias. PCI was identified using ICD-10-PCS codes for coronary artery dilation and extirpation procedures (Supplementary Table S1). The index date was defined as the date of first AMI diagnosis during the study period. Patients were required to have RDW measured on the index date and documented sex. For patients with multiple AMI episodes during the study period, only the first event was included. Patients were followed until 31 December 2025, or the occurrence of death or outcome events. (Supplementary Figure S1).

### 2.3. Exposure Definition (RDW Measurement)

The primary exposure was RDW, measured on the index date of AMI diagnosis. Patients were categorized as high RDW (≥13.5%) or low RDW (<13.5%). This cutoff value approximates the median RDW in our study population. The 13.5% threshold has been used as a stratification point in prior cardiovascular studies examining RDW and clinical outcomes [21,22]. Detailed query criteria for both cohorts are provided in Supplementary Tables S1 and S2.

### 2.4. Outcome Definitions

The primary outcome was all-cause mortality within 1 year after the index AMI. Mortality was ascertained from death records in the electronic health records, which may include data from vital statistics and administrative sources [20]. Secondary outcomes included MACE, defined as a composite of recurrent myocardial infarction, cerebrovascular accident, heart failure hospitalization, cardiogenic shock, and cardiac arrhythmia. Individual components were assessed separately: heart failure (ICD-10-CM I50), cardiogenic shock (R57.0), recurrent AMI (I21, I22), cerebrovascular accident (I60–I63), ventricular tachycardia/ventricular fibrillation (I47.2, I49.0), and cardiac arrhythmia (I45, I47–I49). Complete ICD-10-CM code definitions are provided in Supplementary Table S3.

### 2.5. Covariates and Baseline Characteristics

Baseline characteristics assessed during the 365 days preceding the index AMI included demographic variables (age, sex, race, ethnicity) and 26 comorbidities. Comorbidities encompassed cardiovascular diseases (hypertension, ischemic heart disease, heart failure, cerebrovascular disease, conduction disorders), cardiometabolic conditions (diabetes, dyslipidemia, obesity), chronic organ dysfunction (kidney disease, respiratory disease, liver disease), anemia-related disorders, neoplasms, and neuropsychiatric conditions (dementia, mood and psychotic disorders, substance use disorders, Parkinson’s disease, epilepsy). Complete diagnostic code definitions are in Supplementary Table S3.

### 2.6. Statistical Analysis

Baseline characteristics were reported as mean (standard deviation) for continuous variables and frequency (percentage) for categorical variables. Standardized mean differences (SMD) were used to compare baseline characteristics between groups, with SMD < 0.1 indicating adequate balance.

### 2.7. Propensity Score Matching

To minimize confounding, we performed 1:1 PSM using the TriNetX platform’s built-in algorithm. Propensity scores were estimated using logistic regression with high RDW (≥13.5%) as the outcome and all 38 baseline characteristics (age, sex, race categories, ethnicity categories, and comorbidities) as covariates. Matching was performed using a greedy nearest neighbor algorithm without replacement, with a caliper width of 0.1 pooled standard deviations [16]. The order of matching was randomized to eliminate bias from the nearest neighbor algorithm. Balance between matched cohorts was assessed using SMD, with SMD < 0.1 considered well-balanced.

### 2.8. Primary Analysis

The primary analysis employed a 30-day landmark approach to minimize bias from acute disease severity, peri-procedural complications, and guarantee-time bias [23,24]. Patients who died or experienced outcome events within 30 days after the index AMI were excluded. Among patients surviving beyond 30 days, we compared 1-year outcomes (day 30 to day 365) between high and low RDW groups. Kaplan–Meier survival curves were constructed for time-to-event outcomes, and differences between groups were assessed using the log-rank test. Hazard ratios (HRs) with 95% confidence intervals (CIs) were calculated for time-to-event outcomes using Cox proportional hazards regression. The proportional hazards assumption was assessed using the Schoenfeld residuals test. Moreover, odds ratios (ORs) with 95% CIs were calculated for cumulative event occurrence using logistic regression.

### 2.9. Sensitivity and Subgroup Analyses

We performed five prespecified analyses to assess the robustness of our findings. First, we repeated the analysis without the 30-day landmark (outcomes from day 1 to day 365) to evaluate the impact of early events. Second, to further evaluate whether RDW is an independent predictor of outcomes after adjustment for clinically relevant covariates, we performed a multivariable Cox proportional hazards regression analysis in the full unmatched cohort without applying PSM, using the 30-day landmark approach. The model was adjusted for demographic variables (age, sex, race, and ethnicity) and all 26 prespecified comorbidities, as defined in Section 2.5. Third, to assess the longer-term prognostic value of RDW, we examined 2-year outcomes (day 30 to day 730) using the same propensity score-matched cohorts and analytical approach as the primary analysis. Fourth, to examine whether RDW’s prognostic value was independent of anemia, we stratified patients by baseline hemoglobin concentration (non-anemic: hemoglobin ≥ 12 g/dL; anemic: hemoglobin < 12 g/dL). Within each hemoglobin stratum, we performed 1:1 PSM and repeated the outcome analyses using the 30-day landmark approach. Fifth, to explore whether the prognostic value of RDW differed by AMI type, we performed a subgroup analysis stratifying patients by STEMI (ICD-10-CM codes I21.0–I21.3) and NSTEMI (ICD-10-CM codes I21.4 and I21.9). Within each subgroup, we applied the same 1:1 PSM methodology and repeated the primary outcome analysis using the 30-day landmark approach.

All analyses were conducted using the TriNetX Analytics platform. Two-sided *p*-values < 0.05 were considered statistically significant. Given the exploratory nature of this study and the clinical relevance of all outcomes examined, we did not adjust for multiple comparisons.

## 3. Results

### 3.1. Study Population and Baseline Characteristics

**Figure 1:**
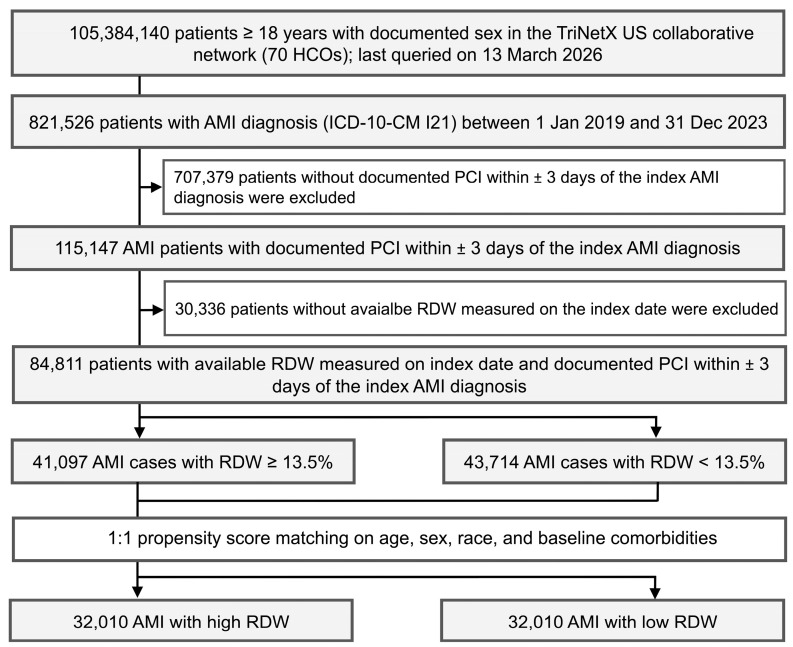
Flow diagram of cohort selection and propensity score matching. AMI: acute myocardial infarction, RDW: red cell distribution width, PCI: percutaneous coronary intervention.

From 1 January 2019, to 31 December 2023, we identified 821,526 adult patients diagnosed with AMI in the TriNetX US Collaborative Network. After applying inclusion criteria, the final cohort comprised 84,811 patients. Of these, 41,097 (48.5%) had high RDW (≥13.5%) and 43,714 (51.5%) had low RDW (<13.5%) (Figure 1).

Before PSM, patients with high RDW were older (mean age 66.3 ± 12.4 years vs. 63.0 ± 12.3 years; SMD 0.266), more likely to be female (35.8% vs. 26.2%; SMD 0.209), and had a higher prevalence of most comorbidities, including heart failure (22.4% vs. 10.9%; SMD 0.313), chronic kidney disease (17.2% vs. 7.9%; SMD 0.284), and anemia-related disorders (Table 1).

After 1:1 PSM, 32,010 patients from each group were successfully matched. The distribution of propensity scores before and after matching demonstrated improved overlap and balance between groups (Supplementary Figure S2). All baseline characteristics were well-balanced with all SMDs < 0.02 (Table 1). The matched cohorts had similar demographic profiles (mean age 65.1–65.2 years, approximately 68% male) and comorbidity burden.

### 3.2. Primary Outcomes: 30-Day Landmark Analysis

**Figure 2:**
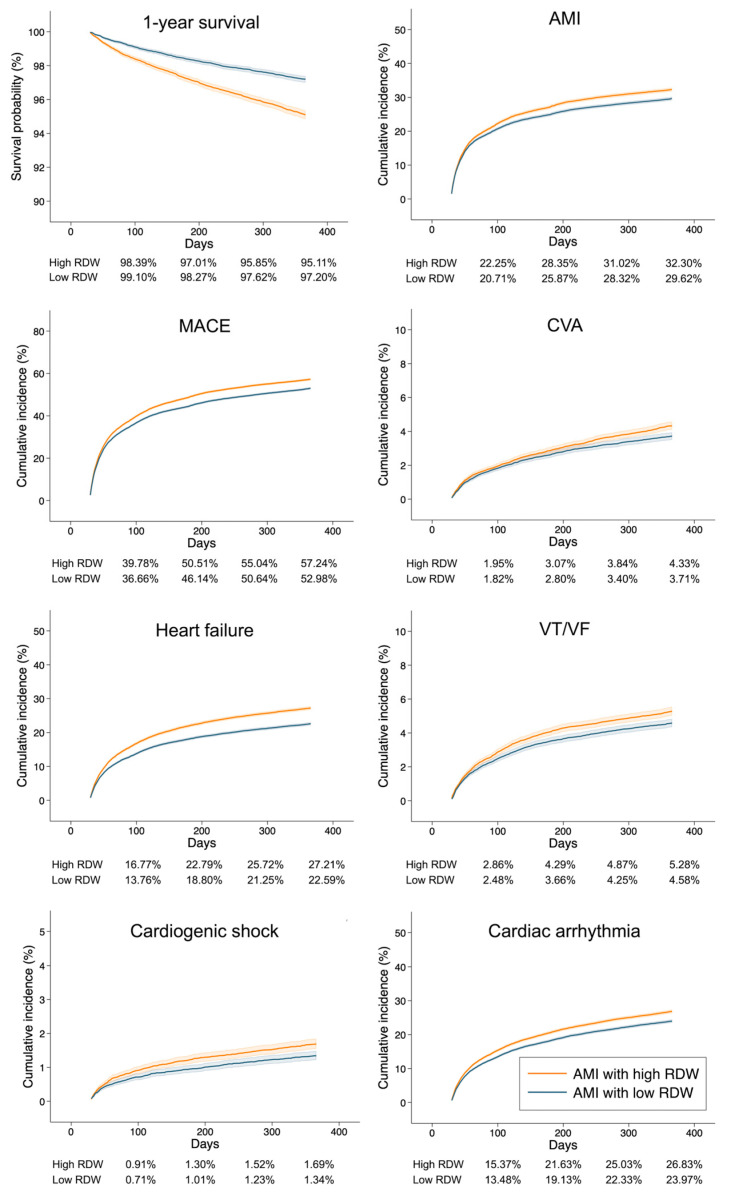
Kaplan–Meier survival and cumulative incidence curves comparing outcomes between high and low RDW groups after acute myocardial infarction. Kaplan–Meier survival and cumulative incidence curves depict clinical outcomes using a 30-day landmark as the primary analytic framework. The analysis included patients who survived and remained at risk at 30 days after the index acute myocardial infarction (AMI) diagnosis, with follow-up commencing on day 30 and extending through day 365. Kaplan–Meier curves illustrate 1-year survival, and cumulative incidence curves show the incidence of recurrent AMI, major adverse cardiovascular events (MACE), cerebrovascular accident (CVA), heart failure, ventricular tachycardia/ventricular fibrillation (VT/VF), cardiogenic shock, and cardiac arrhythmia. Patients were stratified according to red blood cell distribution width (RDW) measured at the index AMI diagnosis (high RDW vs. low RDW). Time is shown in days from the landmark time point. Tabulated values below each panel display the survival probability or cumulative event incidence (%) for each group at Days 100, 200, 300, and 365. Group differences were assessed using the log-rank test; all comparisons were statistically significant (*p* < 0.001), except for cardiogenic shock (*p* = 0.001).

Among propensity score-matched patients surviving beyond 30 days, Kaplan–Meier survival curves and cumulative incidence curves showed consistent separation between groups, with higher event rates in the high RDW group throughout the follow-up period (Figure 2).

Cox proportional hazards regression demonstrated that high RDW (≥13.5%) was significantly associated with increased 1-year all-cause mortality (HR 1.77, 95% CI 1.62–1.93, *p* < 0.001). High RDW was also associated with increased risks of MACE (HR 1.12, 95% CI 1.10–1.15, *p* < 0.001), heart failure (HR 1.24, 95% CI 1.20–1.28, *p* < 0.001), cardiogenic shock (HR 1.26, 95% CI 1.10–1.44, *p* = 0.001), recurrent AMI (HR 1.11, 95% CI 1.07–1.14, *p* < 0.001), cerebrovascular accident (HR 1.16, 95% CI 1.07–1.27, *p* < 0.001), ventricular tachycardia/ventricular fibrillation (HR 1.16, 95% CI 1.07–1.25, *p* < 0.001), and cardiac arrhythmia (HR 1.14, 95% CI 1.10–1.18, *p* < 0.001). The proportional hazards assumption was met for all outcomes (Schoenfeld residuals test *p* > 0.05), except for cerebrovascular accident (*p* = 0.016), suggesting that the hazard ratio for this outcome may vary over time.

**Figure 3:**
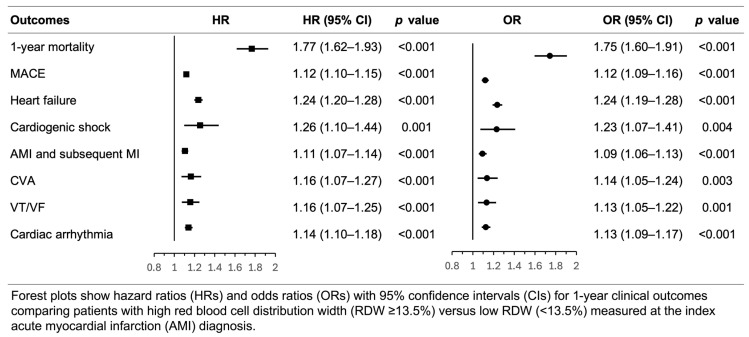
Association between high red blood cell distribution width and 1-year clinical outcomes after acute myocardial infarction (primary 30-day landmark analysis).

Logistic regression analysis for cumulative event occurrence yielded consistent results, with ORs of 1.75 (95% CI 1.60–1.91, *p* < 0.001) for mortality, 1.12 (95% CI 1.09–1.16, *p* < 0.001) for MACE, 1.24 (95% CI 1.19–1.28, *p* < 0.001) for heart failure, 1.23 (95% CI 1.07–1.41, *p* = 0.004) for cardiogenic shock, 1.09 (95% CI 1.06–1.13, *p* < 0.001) for recurrent AMI, 1.14 (95% CI 1.05–1.24, *p* = 0.003) for cerebrovascular accident, 1.13 (95% CI 1.05–1.22, *p* = 0.001) for ventricular tachycardia/ventricular fibrillation, and 1.13 (95% CI 1.09–1.17, *p* < 0.001) for cardiac arrhythmia (Figure 3).

### 3.3. Sensitivity Analyses

**Figure 4:**
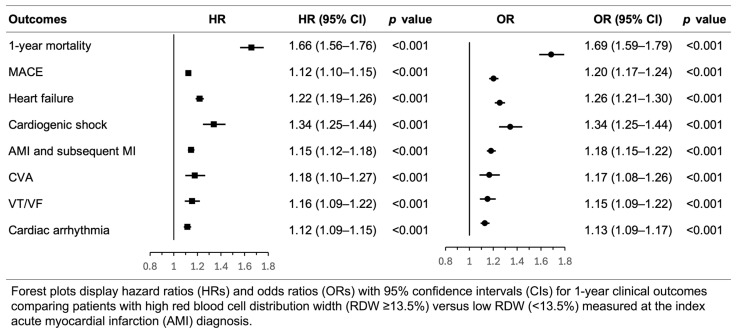
Sensitivity analysis without a 30-day landmark assessing the association between high red blood cell distribution width and 1-year clinical outcomes after acute myocardial infarction.

When outcomes were assessed from day 1 to day 365 (without the 30-day landmark), the associations remained statistically significant but were slightly attenuated compared with the primary analysis. The HR for 1-year mortality was 1.66 (95% CI 1.56–1.76, *p* < 0.001) and OR was 1.69 (95% CI 1.59–1.79, *p* < 0.001). High RDW remained associated with all secondary outcomes (Figure 4, Supplementary Figure S3).

When follow-up was extended to 2 years (day 30 to day 730), high RDW remained significantly associated with increased mortality (HR 1.70, 95% CI 1.59–1.82, *p* < 0.001; OR 1.68, 95% CI 1.57–1.81, *p* < 0.001) and all secondary outcomes. The magnitude of associations remained similar to the 1-year analysis, demonstrating sustained prognostic value of RDW beyond the first year (Supplementary Figure S4).

To confirm the independent prognostic role of RDW after adjustment for clinically relevant covariates, we performed multivariable Cox proportional hazards regression in the full unmatched cohort using the 30-day landmark approach, adjusting for age, sex, race, ethnicity, and all 26 prespecified comorbidities. High RDW remained a significant and independent predictor of 1-year all-cause mortality (adjusted HR 1.88, 95% CI 1.72–2.04, *p* < 0.001) and all secondary outcomes after full covariate adjustment, consistent with the primary PSM-based analysis (Supplementary Figure S5).

### 3.4. Subgroup Analysis by Hemoglobin Status

**Figure 5:**
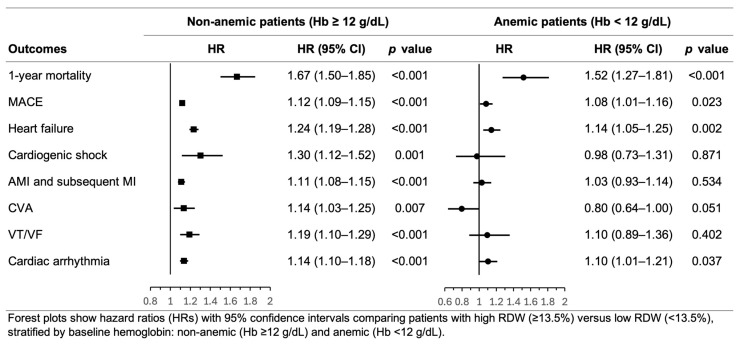
Subgroup analysis of the association between high red blood cell distribution width and 1-year clinical outcomes after acute myocardial infarction, stratified by baseline hemoglobin level.

Among non-anemic patients (hemoglobin ≥12 g/dL), high RDW remained strongly associated with increased 1-year mortality (HR 1.67, 95% CI 1.50–1.85, *p* < 0.001) and all secondary outcomes, including MACE (HR 1.12, 95% CI 1.09–1.15, *p* < 0.001), heart failure (HR 1.24, 95% CI 1.19–1.28, *p* < 0.001), cardiogenic shock (HR 1.30, 95% CI 1.12–1.52, *p* = 0.001), recurrent AMI (HR 1.11, 95% CI 1.08–1.15, *p* < 0.001), cerebrovascular accident (HR 1.14, 95% CI 1.03–1.25, *p* = 0.007), ventricular tachycardia/ventricular fibrillation (HR 1.19, 95% CI 1.10–1.29, *p* < 0.001), and cardiac arrhythmia (HR 1.14, 95% CI 1.10–1.18, *p* < 0.001). Among anemic patients (hemoglobin < 12 g/dL), high RDW remained significantly associated with mortality (HR 1.52, 95% CI 1.27–1.81, *p* < 0.001), MACE (HR 1.08, 95% CI 1.01–1.16, *p* = 0.023), heart failure (HR 1.14, 95% CI 1.05–1.25, *p* = 0.002), and cardiac arrhythmia (HR 1.10, 95% CI 1.01–1.21, *p* = 0.037). However, associations with cardiogenic shock (HR 0.98, 95% CI 0.73–1.31, *p* = 0.871), recurrent AMI (HR 1.03, 95% CI 0.93–1.14, *p* = 0.534), cerebrovascular accident (HR 0.80, 95% CI 0.64–1.00, *p* = 0.051), and ventricular tachycardia/ventricular fibrillation (HR 1.10, 95% CI 0.89–1.36, *p* = 0.402) were not statistically significant (Figure 5).

### 3.5. Subgroup Analysis by Acute Myocardial Infarction Types

To explore whether the prognostic value of RDW differed according to AMI type, we performed a subgroup analysis stratifying patients into STEMI and NSTEMI subgroups. In both subgroups, elevated RDW was consistently associated with increased risk across most primary and secondary outcomes.

Among STEMI patients, high RDW was associated with significantly higher 1-year all-cause mortality (HR 1.95, 95% CI 1.71–2.23, *p* < 0.001) and all secondary outcomes, including MACE (HR 1.12, 95% CI 1.09–1.16, *p* < 0.001), heart failure (HR 1.28, 95% CI 1.22–1.34, *p* < 0.001), cardiogenic shock (HR 1.61, 95% CI 1.34–1.94, *p* < 0.001), recurrent AMI (HR 1.07, 95% CI 1.03–1.11, *p* < 0.001), cerebrovascular accident (HR 1.15, 95% CI 1.02–1.31, *p* = 0.027), ventricular tachycardia/ventricular fibrillation (HR 1.19, 95% CI 1.07–1.31, *p* < 0.001), and cardiac arrhythmia (HR 1.16, 95% CI 1.10–1.21, *p* < 0.001).

Among NSTEMI patients, high RDW was similarly associated with increased 1-year mortality (HR 1.83, 95% CI 1.67–2.05, *p* < 0.001) and most secondary outcomes, including MACE (HR 1.08, 95% CI 1.05–1.11, *p* < 0.001), heart failure (HR 1.30, 95% CI 1.25–1.36, *p* < 0.001), cardiogenic shock (HR 1.32, 95% CI 1.09–1.59, *p* = 0.004), ventricular tachycardia/ventricular fibrillation (HR 1.25, 95% CI 1.13–1.38, *p* < 0.001), and cardiac arrhythmia (HR 1.14, 95% CI 1.09–1.19, *p* < 0.001). However, associations with recurrent AMI (HR 1.04, 95% CI 1.00–1.07, *p* = 0.068) and cerebrovascular accident (HR 1.09, 95% CI 0.97–1.21, *p* = 0.137) did not reach statistical significance in the NSTEMI subgroup (Supplementary Figure S6).

## 4. Discussion

In this large multicenter retrospective cohort study of 84,811 patients with AMI who underwent PCI, we found that elevated RDW (≥13.5%) was independently associated with increased risks of mortality and adverse cardiovascular outcomes. After 1:1 PSM on 38 baseline characteristics, high RDW remained significantly associated with a 77% increased risk of 1-year all-cause mortality (HR 1.77, 95% CI 1.62–1.93) using the 30-day landmark approach. High RDW was also associated with increased risks of MACE, heart failure, cardiogenic shock, recurrent AMI, cerebrovascular accident, and cardiac arrhythmias. These associations were robust across multiple sensitivity analyses, including analysis without the landmark period, extended 2-year follow-up, multivariable Cox proportional hazards regression, and stratification by baseline hemoglobin status and AMI type. Notably, the prognostic value of RDW persisted among non-anemic patients (HR 1.67 for mortality), suggesting that the association is not solely mediated by anemia. To our knowledge, this is the largest propensity score-matched study to date examining the association between RDW and clinical outcomes in patients with AMI.

Accumulating clinical and experimental evidence suggests that the prognostic significance of RDW in AMI reflects a complex interplay among systemic inflammation, oxidative stress, and dysregulated erythropoiesis [13,25]. AMI is increasingly recognized as an acute inflammatory condition characterized by neutrophil and monocyte recruitment during coronary occlusion, regulatory T-cell–mediated macrophage modulation, cytokine release (e.g., interleukin-1 and interleukin-6), and oxidative burst following myocardial injury [26]. These inflammatory and oxidative processes can disrupt bone marrow erythropoiesis by impairing iron metabolism, suppressing erythroid maturation, and promoting the premature release of morphologically heterogeneous red blood cells into the circulation, ultimately resulting in increased RDW [27].

Beyond serving as a passive biomarker, elevated RDW may actively participate in downstream pathophysiological processes. Experimental animal and human studies have shown that increased red blood cell size heterogeneity alters blood rheology, reduces the distance between circulating blood cells and the vascular wall, and increases interactions among red blood cells, platelets, leukocytes, and the endothelium. These changes promote microvascular obstruction, atherothrombosis, and plaque instability [28]. In the setting of AMI, such mechanisms may contribute to persistent myocardial ischemia, impaired reperfusion, adverse vascular remodeling, and subsequent MACE. Taken together, RDW appears to integrate multiple pathological pathways relevant to AMI, including inflammation, oxidative stress, impaired erythropoiesis, and microcirculatory dysfunction, and may function as a pathophysiological amplifier within the inflammation–thrombosis cascade following myocardial infarction.

These mechanistic insights are corroborated by clinical observational data. A retrospective study of 4088 critically ill patients with AMI admitted to the intensive care unit demonstrated a strong association between elevated RDW measured within 24 h before discharge and increased 1-year all-cause mortality. Patients with RDW ≥ 15.9% and 14.8–15.9% exhibited markedly higher mortality rates (34.5% and 22.5%, respectively), compared with only 5.8% among those with RDW < 13.3% [29]. Similarly, in 979 patients with acute coronary syndrome, elevated RDW independently predicted 3-month MACE (adjusted OR 1.36, 95% CI 1.19–1.55) and 1-year mortality (adjusted OR 1.34, 95% CI 1.05–1.71) [30]. Among ST-elevation myocardial infarction patients undergoing PCI, RDW was independently associated with 6-month MACE (OR 2.179, 95% CI 1.250–3.797) and refractory thrombus (OR 8.799, 95% CI 1.240–62.454), highlighting its relevance in thrombotic burden and post-reperfusion outcomes [31].

The prognostic value of RDW extends beyond coronary artery disease to related cardiovascular conditions. A systematic review and meta-analysis by Su et al., encompassing 22 prospective studies and a total of 80,216 patients with coronary artery disease, demonstrated that higher baseline RDW levels were significantly associated with increased all-cause mortality (pooled RR 2.20, 95% CI 1.42–3.39), as well as both fatal (RR 1.80, 95% CI 1.35–2.41) and non-fatal (RR 1.86, 95% CI 1.50–2.31) cardiovascular events [32]. In heart failure populations, RDW consistently predicts disease incidence, severity, and mortality [33]. Furthermore, in population-based cohorts, elevated RDW was independently associated with higher incidence of total stroke (HR 1.31, 95% CI 1.11–1.54) and ischemic stroke (HR 1.32, 95% CI 1.10–1.58) over a mean follow-up of 15 years [34]. These observations are consistent with our findings demonstrating increased heart failure and cerebrovascular events among AMI patients with elevated RDW. While these findings collectively establish the prognostic significance of RDW across the cardiovascular disease spectrum, methodological limitations in the included studies warrant further investigation.

Our study addresses several of these limitations and offers multiple methodological strengths. First, we employed rigorous 1:1 PSM on 38 baseline characteristics to minimize confounding—an approach used in few prior RDW studies [16,17]. Second, our 30-day landmark analysis specifically addresses immortal time bias and guarantee-time bias [23,24], methodological concerns not systematically addressed in earlier studies. Third, we conducted stratified analyses by hemoglobin status, directly testing whether RDW’s prognostic value is independent of anemia—a question that remained unresolved in prior meta-analyses. Our hemoglobin-stratified analyses demonstrated that RDW remained a significant predictor of mortality in non-anemic patients (HR 1.67), providing definitive evidence that the association is not solely mediated by anemia. Fourth, comprehensive sensitivity analyses, including extended 2-year follow-up and analysis without the landmark period, confirmed the robustness and durability of RDW’s prognostic associations across multiple analytical frameworks. Finally, our substantially larger sample size of 84,811 patients from 70 geographically diverse US healthcare organizations provides more precise effect estimates and broader generalizability to real-world clinical practice than prior studies. Furthermore, subgroup analyses stratified by AMI type demonstrated that the prognostic value of elevated RDW was consistent across both STEMI and NSTEMI patients. Elevated RDW was significantly associated with increased 1-year mortality in STEMI (HR 1.95, 95% CI 1.71–2.23) and NSTEMI (HR 1.83, 95% CI 1.67–2.05) subgroups, with broadly consistent findings across secondary outcomes in both groups. These results suggest that the independent prognostic role of RDW in AMI patients undergoing PCI is not restricted to a particular infarction subtype, further supporting its potential utility as a universal risk stratification tool in this population.

Despite these methodological strengths, several limitations warrant consideration. First, TriNetX aggregates data from HCOs across multiple regions with varying healthcare systems, clinical guidelines, and coding practices, potentially introducing heterogeneity. Second, events occurring outside participating HCOs (home deaths, non-affiliated outpatient care) are not captured, and important clinical details, including coronary disease severity, functional status, and cause-specific mortality, are often incomplete. Third, as a retrospective observational study, residual confounding and selection bias cannot be fully eliminated despite PSM. Fourth, both AMI diagnosis and clinical outcomes were ascertained using ICD-10-CM administrative codes derived from electronic health records. Although ICD-10-CM coding has demonstrated reasonable validity for cardiovascular diagnoses in administrative databases, it is subject to variability in clinical documentation practices across institutions and may not capture all clinically relevant nuances, potentially introducing misclassification bias. Future studies incorporating biomarker-confirmed AMI diagnoses and adjudicated clinical endpoints would provide a stronger evidentiary foundation. Fifth, we assessed RDW only at index AMI and could not evaluate serial measurements, which may provide additional prognostic information. Indeed, Xiao et al. demonstrated that dynamic changes in RDW during hospitalization independently predicted MACE after PCI in patients with unstable angina pectoris [35], suggesting that serial RDW monitoring beyond the index measurement warrants further investigation as a direction for future research. Sixth, the TriNetX federated platform does not support ROC curve generation or AUC computation; formal evaluation of the discriminative accuracy of RDW therefore awaits future studies with access to individual-level data.

Notwithstanding these limitations, our findings have important clinical and translational implications. Several validated risk scores, such as GRACE, Killip classification, and TIMI scores, are widely used for risk stratification in acute coronary syndromes [3,4,36]. However, these tools often require multiple clinical variables that may not be immediately available at presentation. RDW offers complementary value as a readily accessible, cost-effective parameter routinely measured during acute care. Furthermore, Liang et al. recently demonstrated that RDW, together with residual cholesterol and body mass index, constitutes an integrated set of risk factors for premature acute coronary syndrome, further supporting the concept that RDW contributes incremental prognostic value within a multiparametric cardiovascular risk profile [37]. Our findings suggest that RDW, when integrated with existing risk assessment tools, may enhance early prognostic assessment in AMI patients undergoing PCI. While RDW provides a practical hematologic complement to clinical risk scores, it should be interpreted alongside—rather than in place of—advanced imaging evaluation. Cardiac magnetic resonance with late gadolinium enhancement offers mechanistic insights that biomarkers cannot capture, including infarct size, transmural extent of myocardial fibrosis, and microvascular obstruction, each carrying independent prognostic significance after AMI [38]. RDW may therefore be most valuable as a first-line tool for identifying high-risk patients who warrant further comprehensive assessment. However, prospective validation studies are needed to determine whether RDW-guided risk stratification translates into improved clinical decision-making and patient outcomes. Future research should also explore whether interventions targeting the underlying pathophysiological processes reflected by elevated RDW, such as inflammation, oxidative stress, or impaired erythropoiesis, can improve outcomes in high-risk AMI patients. Additionally, prospective studies with access to individual-level data should formally evaluate the discriminative accuracy and incremental prognostic value of RDW when added to established risk scores such as GRACE and TIMI. Such investigations would help determine whether RDW represents a modifiable risk factor or solely a prognostic marker.

## 5. Conclusions

In this large propensity score-matched cohort of 84,811 AMI patients undergoing PCI, elevated RDW (≥13.5%) was independently associated with a 77% increased risk of 1-year all-cause mortality and increased risks of MACE, heart failure, cardiogenic shock, and cerebrovascular events. Importantly, RDW’s prognostic value persisted among non-anemic patients (HR 1.67 for mortality), suggesting that its association with outcomes is not solely mediated by anemia. These findings suggest that RDW, as a readily available and inexpensive biomarker, may complement existing prognostic tools such as GRACE, TIMI, and Killip scores to support early risk stratification in AMI patients undergoing PCI. Prospective validation studies are needed to confirm whether RDW-guided risk stratification translates into improved clinical outcomes.

## Figures and Tables

**Table 1:** Baseline Characteristics of AMI with High and Low RDW Cohort Before and After PSM.

	Before PSM			After PSM		
	Participants, No. (%)			Participants, No. (%)		
Characteristic	AMI with high RDW (n = 41,097)	AMI with low RDW (n = 43,714)	SMD	AMI with high RDW (n = 32,010)	AMI with low RDW (n = 32,010)	SMD
Age at index, mean (SD), y	66.3 (12.4)	63.0 (12.3)	0.266	65.1 (12.3)	65.2 (12.1)	0.013
Sex						
Male	26,388 (64.2%)	32,270 (73.8%)	0.209	21,933 (68.5%)	21,681 (67.7%)	0.017
Female	14,709 (35.8%)	11,444 (26.2%)	0.209	10,077 (31.5%)	10,329 (32.3%)	0.017
Ethnicity						
Hispanic or Latino	1956 (4.8%)	2264 (5.2%)	0.019	1619 (5.1%)	1588 (5.0%)	0.004
Not Hispanic or Latino	35,684 (86.8%)	37,085 (84.8%)	0.057	27,538 (86.0%)	27,602 (86.2%)	0.006
Unknown Ethnicity	3457 (8.4%)	4365 (10.0%)	0.054	2853 (8.9%)	2820 (8.8%)	0.004
Race						
White	30,916 (75.2%)	35,348 (80.9%)	0.136	25,512 (79.7%)	25,565 (79.9%)	0.004
Black or African American	5824 (14.2%)	2771 (6.3%)	0.260	2789 (8.7%)	2760 (8.6%)	0.003
Asian	1179 (2.9%)	2014 (4.6%)	0.092	1067 (3.3%)	1048 (3.3%)	0.003
Native Hawaiian or Other Pacific Islander	304 (0.7%)	417 (1.0%)	0.023	260 (0.8%)	248 (0.8%)	0.004
American Indian or Alaska Native	164 (0.4%)	147 (0.3%)	0.010	129 (0.4%)	125 (0.4%)	0.002
Other ^a^	1334 (3.2%)	1538 (3.5%)	0.015	1113 (3.5%)	1082 (3.4%)	0.005
Unknown Race	1376 (3.3%)	1479 (3.4%)	0.002	1140 (3.6%)	1182 (3.7%)	0.007
Comorbidities						
Ischemic heart diseases	23,471 (57.1%)	21,567 (49.3%)	0.156	16,469 (51.5%)	16,403 (51.2%)	0.004
Hypertension	23,368 (56.9%)	20,252 (46.3%)	0.212	16,055 (50.2%)	15,995 (50.0%)	0.004
Dyslipidemia	20,703 (50.4%)	19,129 (43.8%)	0.133	14,541 (45.4%)	14,421 (45.1%)	0.008
Diabetes mellitus	12,298 (29.9%)	9181 (21.0%)	0.206	7710 (24.1%)	7695 (24.0%)	0.001
Heart failure	9221 (22.4%)	4771 (10.9%)	0.313	4538 (14.2%)	4527 (14.1%)	0.001
Mental and behavioral disorders due to psychoactive substance use	7779 (18.9%)	6578 (15.0%)	0.103	5284 (16.5%)	5275 (16.5%)	0.001
Gastro-esophageal reflux disease	8099 (19.7%)	6352 (14.5%)	0.138	5263 (16.4%)	5057 (15.8%)	0.018
Overweight, obesity and other hyper alimentation	7049 (17.2%)	5348 (12.2%)	0.139	4396 (13.7%)	4342 (13.6%)	0.005
Chronic lower respiratory diseases	7137 (17.4%)	4317 (9.9%)	0.220	4072 (12.7%)	3964 (12.4%)	0.010
Chronic kidney disease	7072 (17.2%)	3448 (7.9%)	0.284	3381 (10.6%)	3290 (10.3%)	0.009
Neoplasms	5647 (13.7%)	4392 (10.0%)	0.114	3657 (11.4%)	3619 (11.3%)	0.004
Mood [affective] disorders	4829 (11.8%)	3550 (8.1%)	0.122	3045 (9.5%)	2965 (9.3%)	0.009
Aplastic and other anemias and other bone marrow failure syndromes	5988 (14.6%)	2350 (5.4%)	0.311	2418 (7.6%)	2309 (7.2%)	0.013
Cerebrovascular diseases	3716 (9.0%)	2330 (5.3%)	0.144	2122 (6.6%)	2095 (6.5%)	0.003
Atrioventricular and left bundle-branch block	3408 (8.3%)	2199 (5.0%)	0.131	1921 (6.0%)	1922 (6.0%)	0.000
Other conduction disorders	2881 (7.0%)	2003 (4.6%)	0.104	1711 (5.3%)	1662 (5.2%)	0.007
Chronic liver disease	2040 (5.0%)	1336 (3.1%)	0.097	1179 (3.7%)	1123 (3.5%)	0.009
Syncope and collapse	1690 (4.1%)	1263 (2.9%)	0.067	1028 (3.2%)	1018 (3.2%)	0.002
Nutritional anemias	2606 (6.3%)	754 (1.7%)	0.236	845 (2.6%)	748 (2.3%)	0.019
Unspecified dementia	593 (1.4%)	316 (0.7%)	0.070	305 (1.0%)	294 (0.9%)	0.004
Epilepsy and recurrent seizures	482 (1.2%)	383 (0.9%)	0.029	307 (1.0%)	299 (0.9%)	0.003
Parkinson’s disease	259 (0.6%)	193 (0.4%)	0.026	174 (0.5%)	159 (0.5%)	0.007
Schizophrenia, schizotypal, delusional, and other non-mood psychotic disorders	274 (0.7%)	161 (0.4%)	0.042	152 (0.5%)	143 (0.4%)	0.004
Alzheimer’s disease	172 (0.4%)	124 (0.3%)	0.023	106 (0.3%)	104 (0.3%)	0.001
Hemolytic anemias	196 (0.5%)	69 (0.2%)	0.057	85 (0.3%)	67 (0.2%)	0.012
Vascular dementia	158 (0.4%)	83 (0.2%)	0.036	72 (0.2%)	75 (0.2%)	0.002

Abbreviations: AMI, acute myocardial infarction; RDW, red cell distribution width; PSM, propensity score matching; SMD, standardized mean difference. ^a^ Includes multiracial and any other race not specified.

## Data Availability

The data that support the findings of this study were obtained from the TriNetX US Collaborative Network. Restrictions apply to the availability of these data, which were accessed under license and data use agreements, and therefore are not publicly available. Data are available from TriNetX (https://trinetx.com, last accessed on 24 February 2026) for researchers who meet the criteria for access to confidential data and have appropriate institutional agreements in place. Aggregate results and study protocols are available from the corresponding author upon reasonable request.

## Supplementary Materials

The following supporting information can be downloaded at: https://www.mdpi.com/article/10.3390/jcm15062432/s1, Supplementary Figure S1: Schematic diagram of the study design, exposure assessment, and outcome ascertainment.; Supplementary Figure S2: Distribution of propensity scores before and after propensity score matching.; Supplementary Figure S3: Kaplan–Meier survival and cumulative incidence curves for sensitivity analysis comparing outcomes between high and low RDW groups after acute myocardial infarction.; Supplementary Figure S4: Association between high red blood cell distribution width and 2-year clinical outcomes after acute myocardial infarction: sensitivity analysis with extended follow-up.; Supplementary Figure S5: Sensitivity analysis using a multivariable Cox proportional hazards model with a 30-day landmark to assess the association between high red blood cell distribution width and 1-year clinical outcomes after acute myocardial infarction.; Supplementary Figure S6: Subgroup analysis of the association between high red blood cell distribution width and 1-year clinical outcomes after acute myocardial infarction according to myocardial infarction type (STEMI vs. NSTEMI).; Supplementary Table S1: Query Criteria for Cohort 1 (AMI cases with RDW ≥ 13.5%).; Supplementary Table S2: Query Criteria for Cohort 2 (AMI cases with RDW < 13.5%).; Supplementary Table S3: ICD-10-CM code definition of comorbidities and outcomes.

## Abbreviations

The following abbreviations are used in this manuscript:

AMI	Acute myocardial infarction
CI	Confidence interval
HCO	Healthcare organizations
HR	Hazard ratio
OR	Odds ratio
PSM	Propensity score matching
PCI	Percutaneous coronary intervention
MACE	Major adverse cardiovascular events
RDW	Red blood cell distribution width
SMD	Standardized mean differences
