# Supplementary material for: Elevated Red Blood Cell Distribution Width Predicts Mortality and Major Adverse Cardiovascular Events After Acute Myocardial Infarction: A Large Propensity Score-Matched Cohort Study
Source: J Clin Med. 2026 Mar 22;15(6):2432. doi: 10.3390/jcm15062432 (PMC13027959; doi:10.3390/jcm15062432)
Supplement: Supplementary file 1 [file jcm-15-02432-s001.zip › jcm-4195792-supplementary.pdf]

Supplementary Material

Table of Contents

**Supplementary Figure S1.** Schematic diagram of the study design, exposure assessment, and outcome ascertainment.	Page 2
**Supplementary Figure S2.** Distribution of propensity scores before and after propensity score matching.	Page 3
**Supplementary Figure S3.** Kaplan–Meier survival and cumulative incidence curves for sensitivity analysis comparing outcomes between high and low RDW groups after acute myocardial infarction.	Page 4
**Supplementary Figure S4.** Association between high red blood cell distribution width and 2-year clinical outcomes after acute myocardial infarction: sensitivity analysis with extended follow-up.	Page 5
**Supplementary Figure S5.** Sensitivity analysis using a multivariable Cox proportional hazards model with a 30-day landmark to assess the association between high red blood cell distribution width and 1-year clinical outcomes after acute myocardial infarction.	Page 6
**Supplementary Figure S6.** Subgroup analysis of the association between high red blood cell distribution width and 1-year clinical outcomes after acute myocardial infarction according to myocardial infarction type (STEMI vs NSTEMI).	Page 7
**Supplementary Table S1.** Query Criteria for Cohort 1 (AMI cases with RDW ≥ 13.5%)	Page 8–16
**Supplementary Table S2.** Query Criteria for Cohort 2 (AMI cases with RDW < 13.5%)	Page 17–25
**Supplementary Table S3.** ICD-10-CM code definition of comorbidities and outcomes.	Page 26

Supplementary Figure S1. Schematic diagram of the study design, exposure assessment, and outcome ascertainment.

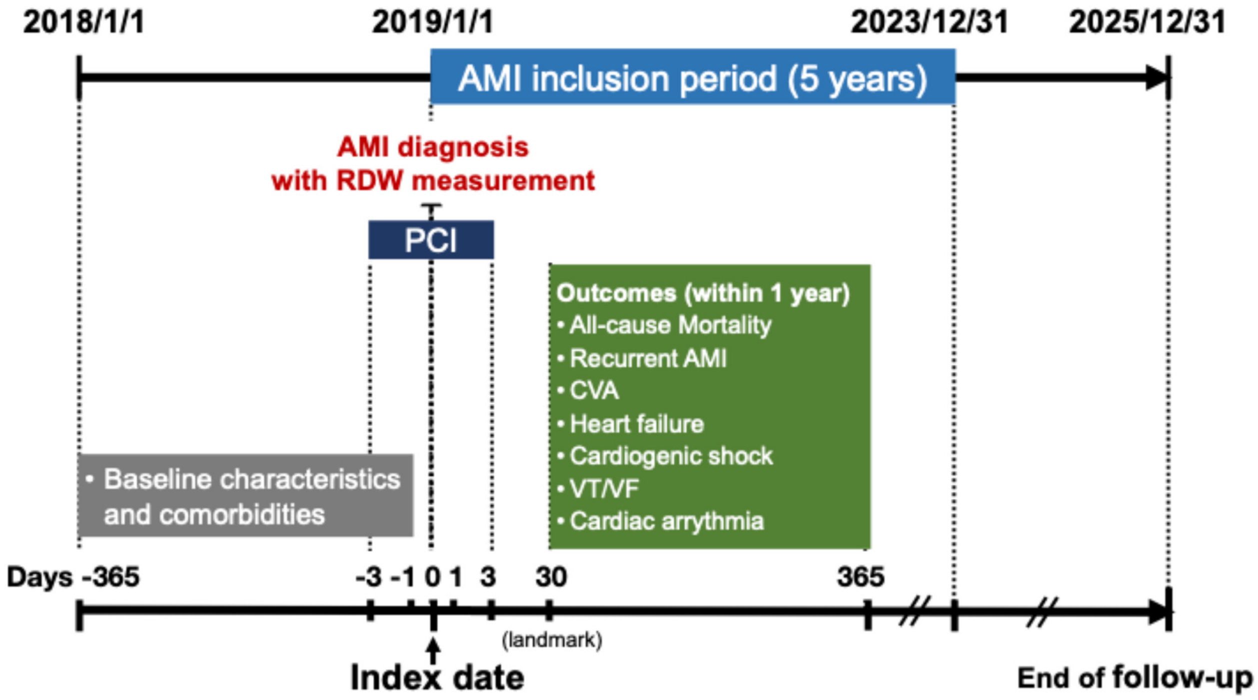

This figure depicts the study timeline and analytic framework. Patients diagnosed with acute myocardial infarction (AMI) between January 1, 2019, and December 31, 2023 were included. Red blood cell distribution width (RDW) was measured on the index date of AMI diagnosis. Percutaneous coronary intervention (PCI) performed within ±3 days of the index date was included to account for inter-facility transfer and potential coding delays. Baseline demographic characteristics and comorbidities were assessed during the 365 days preceding the index date. Clinical outcomes—including all-cause mortality, recurrent AMI, cerebrovascular accident (CVA), heart failure, cardiogenic shock, ventricular tachycardia/ventricular fibrillation (VT/VF), and cardiac arrhythmia—were evaluated using a 30-day landmark as the primary analytic framework, with follow-up from day 30 to 1 year after the index date. This landmark approach was used to minimize bias from early post-AMI events related to acute disease severity, peri-procedural complications, and reverse causation.

Supplementary Figure S2. Distribution of propensity scores before and after propensity score matching.

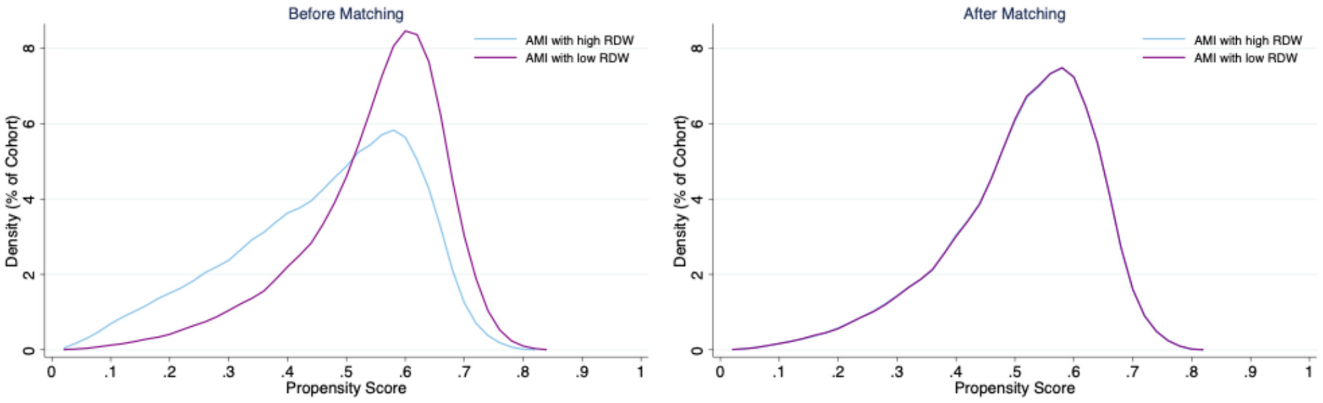

Density plots illustrate the distribution of propensity scores for patients with acute myocardial infarction (AMI) stratified by red blood cell distribution width (RDW) status (high RDW vs low RDW) before and after 1:1 propensity score matching. The left panel shows the propensity score distributions before matching, demonstrating substantial imbalance between groups, whereas the right panel shows the distributions after matching, indicating improved overlap and covariate balance between the high and low RDW cohorts. Propensity scores were estimated using baseline demographic characteristics and comorbidities.

Propensity score matching was performed using 38 baseline characteristics. Demographic variables included age at index, sex, ethnicity, and race. Clinical comorbidities included hypertensive diseases, diabetes mellitus, disorders of lipoprotein metabolism, heart failure, chronic kidney disease, liver diseases, chronic lower respiratory diseases, ischemic heart diseases, cerebrovascular diseases, conduction disorders, syncope, dementia-related diagnoses, psychiatric disorders, malignancies, obesity-related conditions, epilepsy, and anemia-related disorders.

Supplementary Figure S3. Kaplan–Meier survival and cumulative incidence curves for sensitivity analysis comparing outcomes between high and low RDW groups after acute myocardial infarction.

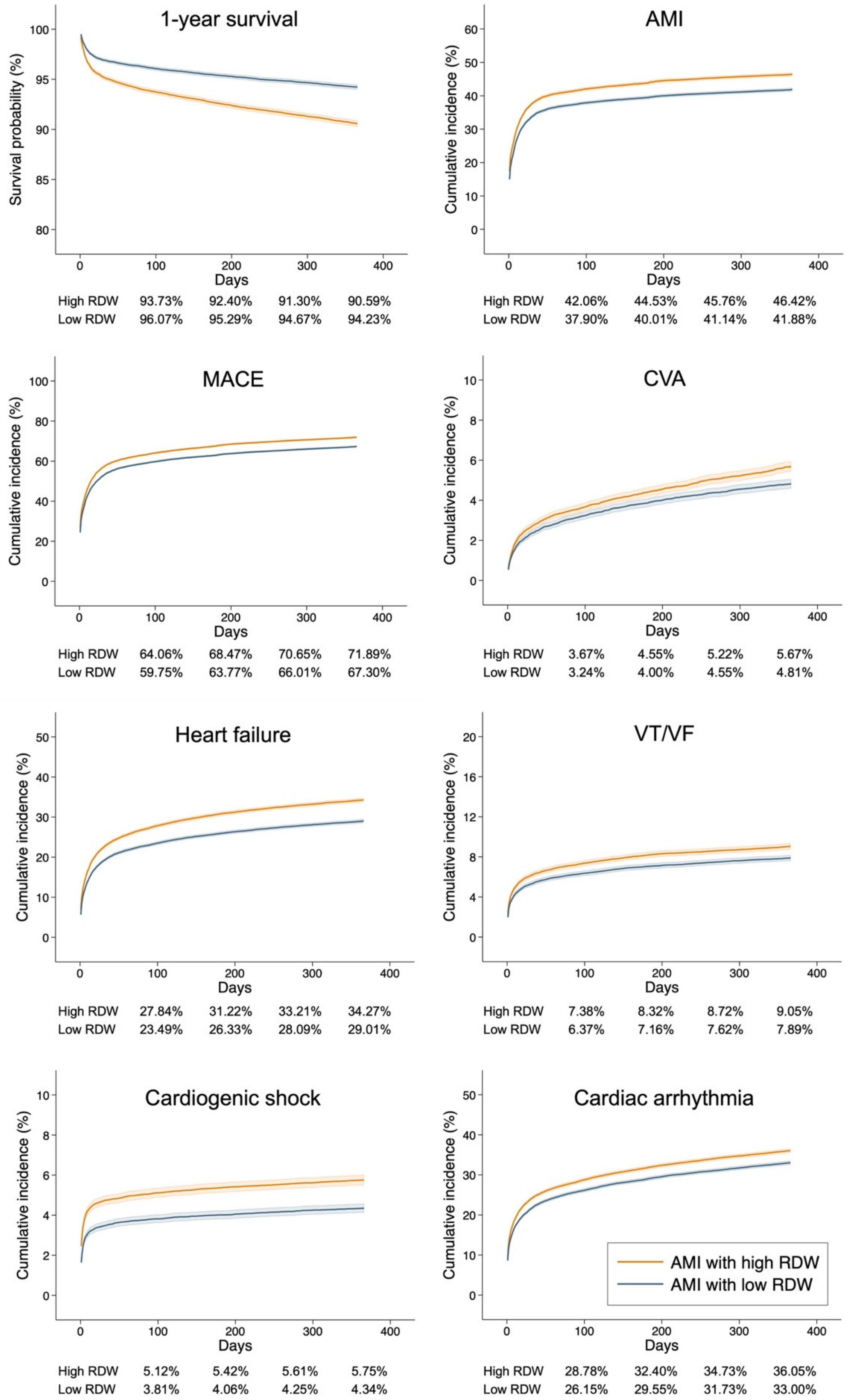

Kaplan–Meier survival and cumulative incidence curves depict clinical outcomes in a sensitivity analysis in which follow-up commenced 1 day after the index acute myocardial infarction (AMI) diagnosis. Kaplan–Meier curves illustrate 1-year survival, and cumulative incidence curves show

the incidence of major adverse cardiovascular events (MACE), heart failure, cardiogenic shock, recurrent AMI, cerebrovascular accident (CVA), ventricular tachycardia/ventricular fibrillation (VT/VF), and cardiac arrhythmia during follow-up. Patients were stratified according to red blood cell distribution width (RDW) measured at the index AMI diagnosis (high RDW vs low RDW). Time is shown in days from day 1 after the index date. Tabulated values below each panel display the survival probability or cumulative event incidence (%) for each group at Days 100, 200, 300, and 365. Group differences were assessed using the log-rank test, and all comparisons were statistically significant ($p < 0.001$).

Supplementary Figure S4. Association between high red blood cell distribution width and 2-year clinical outcomes after acute myocardial infarction: sensitivity analysis with extended follow-up.

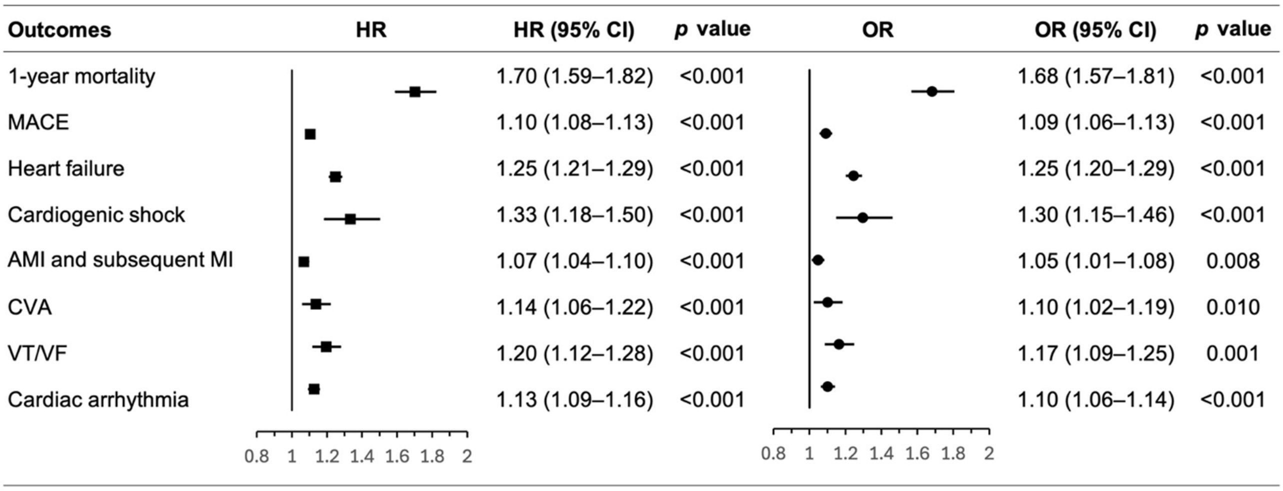

Forest plots show hazard ratios (HRs) and odds ratios (ORs) with 95% confidence intervals (CIs) for 2-year clinical outcomes comparing patients with high red blood cell distribution width (RDW ≥13.5%) versus low RDW (<13.5%) measured at the index acute myocardial infarction (AMI) diagnosis. This sensitivity analysis employed a 30-day landmark approach, including only patients who survived and remained at risk at 30 days after the index AMI diagnosis, with follow-up commencing on day 30 and extending through day 730. Cohorts were balanced using 1:1 propensity score matching on demographics and baseline comorbidities. Vertical line at HR = 1.0 indicates no association.

MACE: major adverse cardiovascular events, AMI: acute myocardial infarction, CVA: cerebrovascular accident, VT/VF: ventricular tachycardia/ventricular fibrillation.

Supplementary Figure S5. Sensitivity analysis using a multivariable Cox proportional hazards model with a 30-day landmark to assess the association between high red blood cell distribution width and 1-year clinical outcomes after acute myocardial infarction.

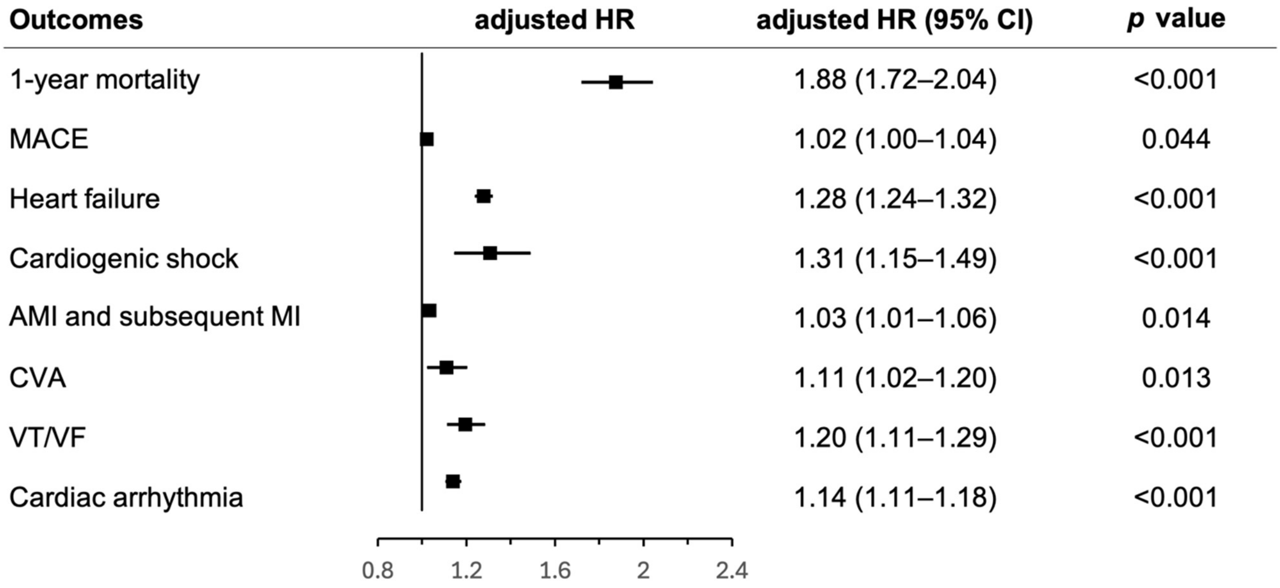

Outcomes	adjusted HR (95% CI)	*p* value
1-year mortality	1.88 (1.72–2.04)	<0.001
MACE	1.02 (1.00–1.04)	0.044
Heart failure	1.28 (1.24–1.32)	<0.001
Cardiogenic shock	1.31 (1.15–1.49)	<0.001
AMI and subsequent MI	1.03 (1.01–1.06)	0.014
CVA	1.11 (1.02–1.20)	0.013
VT/VF	1.20 (1.11–1.29)	<0.001
Cardiac arrhythmia	1.14 (1.11–1.18)	<0.001

Forest plots display adjusted hazard ratios (HRs) with 95% confidence intervals (CIs) for 1-year clinical outcomes comparing patients with high red blood cell distribution width (RDW ≥13.5%) versus low RDW (<13.5%) measured at the index acute myocardial infarction (AMI) diagnosis. This sensitivity analysis was performed using a multivariable Cox proportional hazards model adjusting for demographic variables and comorbidities and applying a 30-day landmark. Outcomes included all-cause mortality, major adverse cardiovascular events (MACE), heart failure, cardiogenic shock, recurrent AMI, cerebrovascular accident (CVA), ventricular tachycardia/ventricular fibrillation (VT/VF), and cardiac arrhythmia.
A vertical reference line at unity (HR = 1.0) indicates no association.

Supplementary Figure S6. Subgroup analysis of the association between high red blood cell distribution width and 1-year clinical outcomes after acute myocardial infarction according to myocardial infarction type (STEMI vs NSTEMI).

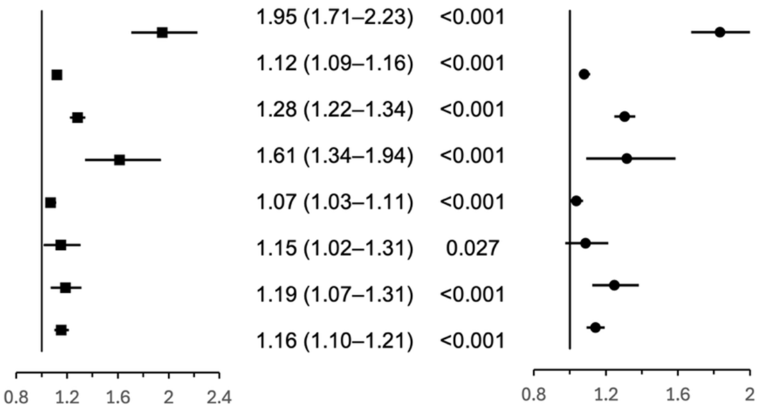

	STEMI			NSTEMI		
Outcomes	HR	HR (95% CI)	*p* value	HR	HR (95% CI)	*p* value
1-year mortality		1.95 (1.71–2.23)	<0.001		1.83 (1.67–2.05)	<0.001
MACE		1.12 (1.09–1.16)	<0.001		1.08 (1.05–1.11)	<0.001
Heart failure		1.28 (1.22–1.34)	<0.001		1.30 (1.25–1.36)	<0.001
Cardiogenic shock		1.61 (1.34–1.94)	<0.001		1.32 (1.09–1.59)	0.004
AMI and subsequent MI		1.07 (1.03–1.11)	<0.001		1.04 (1.00–1.07)	0.068
CVA		1.15 (1.02–1.31)	0.027		1.09 (0.97–1.21)	0.137
VT/VF		1.19 (1.07–1.31)	<0.001		1.25 (1.13–1.38)	<0.001
Cardiac arrhythmia		1.16 (1.10–1.21)	<0.001		1.14 (1.09–1.19)	<0.001

Forest plots show hazard ratios (HRs) with 95% confidence intervals comparing patients with high RDW (≥13.5%) versus low RDW (<13.5%), stratified by myocardial infarction type (STEMI and NSTEMI). A 30-day landmark approach was applied, with follow-up from day 30 to day 365 after index acute myocardial infarction. Cohorts were balanced using 1:1 propensity score matching on demographics and baseline comorbidities. Vertical line at HR = 1.0 indicates no association. *p* values from two-sided tests. MACE: major adverse cardiovascular events, AMI: acute myocardial infarction, CVA: cerebrovascular accident, VT/VF: ventricular tachycardia/ventricular fibrillation. STEMI: ST-segment elevation myocardial infarction, NSTEMI: non-ST-segment elevation myocardial infarction.

Supplementary Table S1. Query Criteria for Cohort 1 (AMI cases with RDW ≥ 13.5%)

This query was run on the network US Collaborative Network with 70 HCO(s) queried and 70 HCO(s) responded. A total of 51 provider(s) responded with patients. The final cohort included 41,097 patients who matched the query criteria listed in the table below.

**Ungrouped terms**				
must have		demographics	Age	Age (at least 18 years (most recent occurrence))
	and any of	demographics	UMLS:HL7V3.0:Gender: M	Male
		demographics	UMLS:HL7V3.0:Gender: F	Female
**Group 1**				
**AMI**				
must have		diagnosis	UMLS:ICD10CM:I21	Acute myocardial infarction
date constraint		The terms in this group occurred between Jan 1, 2019 and Dec 31, 2023		
**Group 2**				
**Group 2A AMI**				
must have		diagnosis	UMLS:ICD10CM:I21	Acute myocardial infarction
date constraint		The terms in this group occurred at any time		
event relationship		Any instance of PCI occurred within 3 days before or up to 3 days after the first instance of AMI		
**Group 2B PCI**				
must have	any of	procedure	UMLS:ICD10PCS:02F0	Heart And Great Vessels / Fragmentation / Coronary Artery, One Artery
		procedure	UMLS:ICD10PCS:02F1	Heart And Great Vessels / Fragmentation / Coronary Artery, Two Arteries
		procedure	UMLS:ICD10PCS:02F2	Heart And Great Vessels / Fragmentation / Coronary Artery, Three Arteries
		procedure	UMLS:ICD10PCS:02F3	Heart And Great Vessels / Fragmentation / Coronary Artery, Four Or More Arteries
		procedure	UMLS:ICD10PCS:0270346	Dilation of Coronary Artery, One Artery, Bifurcation, with Drug-eluting Intraluminal Device, Percutaneous Approach
		procedure	UMLS:ICD10PCS:027034Z	Dilation of Coronary Artery, One Artery with Drug-eluting Intraluminal Device, Percutaneous Approach
		procedure	UMLS:ICD10PCS:0270356	Dilation of Coronary Artery, One Artery, Bifurcation, with Two Drug-eluting Intraluminal Devices, Percutaneous Approach
		procedure	UMLS:ICD10PCS:027035Z	Dilation of Coronary Artery, One Artery with Two Drug-eluting Intraluminal Devices, Percutaneous Approach
		procedure	UMLS:ICD10PCS:0270366	Dilation of Coronary Artery, One Artery, Bifurcation, with Three Drug-eluting Intraluminal Devices, Percutaneous Approach
		procedure	UMLS:ICD10PCS:027036Z	Dilation of Coronary Artery, One Artery with Three Drug-eluting Intraluminal Devices, Percutaneous Approach

procedure	UMLS:ICD10PCS:0270376	Dilation of Coronary Artery, One Artery, Bifurcation, with Four or More Drug-eluting Intraluminal Devices, Percutaneous Approach
procedure	UMLS:ICD10PCS:027037Z	Dilation of Coronary Artery, One Artery with Four or More Drug-eluting Intraluminal Devices, Percutaneous Approach
procedure	UMLS:ICD10PCS:02703D6	Dilation of Coronary Artery, One Artery, Bifurcation, with Intraluminal Device, Percutaneous Approach
procedure	UMLS:ICD10PCS:02703E6	Dilation of Coronary Artery, One Artery, Bifurcation, with Two Intraluminal Devices, Percutaneous Approach
procedure	UMLS:ICD10PCS:02703DZ	Dilation of Coronary Artery, One Artery with Intraluminal Device, Percutaneous Approach
procedure	UMLS:ICD10PCS:02703EZ	Dilation of Coronary Artery, One Artery with Two Intraluminal Devices, Percutaneous Approach
procedure	UMLS:ICD10PCS:02703F6	Dilation of Coronary Artery, One Artery, Bifurcation, with Three Intraluminal Devices, Percutaneous Approach
procedure	UMLS:ICD10PCS:02703FZ	Dilation of Coronary Artery, One Artery with Three Intraluminal Devices, Percutaneous Approach
procedure	UMLS:ICD10PCS:02703G6	Dilation of Coronary Artery, One Artery, Bifurcation, with Four or More Intraluminal Devices, Percutaneous Approach
procedure	UMLS:ICD10PCS:02703GZ	Dilation of Coronary Artery, One Artery with Four or More Intraluminal Devices, Percutaneous Approach
procedure	UMLS:ICD10PCS:02703T6	Dilation of Coronary Artery, One Artery, Bifurcation, with Radioactive Intraluminal Device, Percutaneous Approach
procedure	UMLS:ICD10PCS:02703TZ	Dilation of Coronary Artery, One Artery with Radioactive Intraluminal Device, Percutaneous Approach
procedure	UMLS:ICD10PCS:02703Z6	Dilation of Coronary Artery, One Artery, Bifurcation, Percutaneous Approach
procedure	UMLS:ICD10PCS:02703ZZ	Dilation of Coronary Artery, One Artery, Percutaneous Approach
procedure	UMLS:ICD10PCS:0270446	Dilation of Coronary Artery, One Artery, Bifurcation, with Drug-eluting Intraluminal Device, Percutaneous Endoscopic Approach
procedure	UMLS:ICD10PCS:027044Z	Dilation of Coronary Artery, One Artery with Drug-eluting Intraluminal Device, Percutaneous Endoscopic Approach
procedure	UMLS:ICD10PCS:0270456	Dilation of Coronary Artery, One Artery, Bifurcation, with Two Drug-eluting Intraluminal Devices, Percutaneous Endoscopic Approach
procedure	UMLS:ICD10PCS:027045Z	Dilation of Coronary Artery, One Artery with Two Drug-eluting Intraluminal

		Devices, Percutaneous Endoscopic Approach
procedure	UMLS:ICD10PCS:027046Z	Dilation of Coronary Artery, One Artery with Three Drug-eluting Intraluminal Devices, Percutaneous Endoscopic Approach
procedure	UMLS:ICD10PCS:027047Z	Dilation of Coronary Artery, One Artery with Four or More Drug-eluting Intraluminal Devices, Percutaneous Endoscopic Approach
procedure	UMLS:ICD10PCS:02704D6	Dilation of Coronary Artery, One Artery, Bifurcation, with Intraluminal Device, Percutaneous Endoscopic Approach
procedure	UMLS:ICD10PCS:02704DZ	Dilation of Coronary Artery, One Artery with Intraluminal Device, Percutaneous Endoscopic Approach
procedure	UMLS:ICD10PCS:02704EZ	Dilation of Coronary Artery, One Artery with Two Intraluminal Devices, Percutaneous Endoscopic Approach
procedure	UMLS:ICD10PCS:02704FZ	Dilation of Coronary Artery, One Artery with Three Intraluminal Devices, Percutaneous Endoscopic Approach
procedure	UMLS:ICD10PCS:02704Z6	Dilation of Coronary Artery, One Artery, Bifurcation, Percutaneous Endoscopic Approach
procedure	UMLS:ICD10PCS:02704ZZ	Dilation of Coronary Artery, One Artery, Percutaneous Endoscopic Approach
procedure	UMLS:ICD10PCS:0271346	Dilation of Coronary Artery, Two Arteries, Bifurcation, with Drug-eluting Intraluminal Device, Percutaneous Approach
procedure	UMLS:ICD10PCS:027134Z	Dilation of Coronary Artery, Two Arteries with Drug-eluting Intraluminal Device, Percutaneous Approach
procedure	UMLS:ICD10PCS:0271356	Dilation of Coronary Artery, Two Arteries, Bifurcation, with Two Drug-eluting Intraluminal Devices, Percutaneous Approach
procedure	UMLS:ICD10PCS:027135Z	Dilation of Coronary Artery, Two Arteries with Two Drug-eluting Intraluminal Devices, Percutaneous Approach
procedure	UMLS:ICD10PCS:0271366	Dilation of Coronary Artery, Two Arteries, Bifurcation, with Three Drug-eluting Intraluminal Devices, Percutaneous Approach
procedure	UMLS:ICD10PCS:027136Z	Dilation of Coronary Artery, Two Arteries with Three Drug-eluting Intraluminal Devices, Percutaneous Approach
procedure	UMLS:ICD10PCS:0271376	Dilation of Coronary Artery, Two Arteries, Bifurcation, with Four or More Drug-eluting Intraluminal Devices, Percutaneous Approach
procedure	UMLS:ICD10PCS:027137Z	Dilation of Coronary Artery, Two Arteries with Four or More Drug-eluting Intraluminal Devices, Percutaneous Approach

procedure	UMLS:ICD10PCS:02713D6	Dilation of Coronary Artery, Two Arteries, Bifurcation, with Intraluminal Device, Percutaneous Approach
procedure	UMLS:ICD10PCS:02713DZ	Dilation of Coronary Artery, Two Arteries with Intraluminal Device, Percutaneous Approach
procedure	UMLS:ICD10PCS:02713E6	Dilation of Coronary Artery, Two Arteries, Bifurcation, with Two Intraluminal Devices, Percutaneous Approach
procedure	UMLS:ICD10PCS:02713EZ	Dilation of Coronary Artery, Two Arteries with Two Intraluminal Devices, Percutaneous Approach
procedure	UMLS:ICD10PCS:02713F6	Dilation of Coronary Artery, Two Arteries, Bifurcation, with Three Intraluminal Devices, Percutaneous Approach
procedure	UMLS:ICD10PCS:02713FZ	Dilation of Coronary Artery, Two Arteries with Three Intraluminal Devices, Percutaneous Approach
procedure	UMLS:ICD10PCS:02713G6	Dilation of Coronary Artery, Two Arteries, Bifurcation, with Four or More Intraluminal Devices, Percutaneous Approach
procedure	UMLS:ICD10PCS:02713GZ	Dilation of Coronary Artery, Two Arteries with Four or More Intraluminal Devices, Percutaneous Approach
procedure	UMLS:ICD10PCS:02713T6	Dilation of Coronary Artery, Two Arteries, Bifurcation, with Radioactive Intraluminal Device, Percutaneous Approach
procedure	UMLS:ICD10PCS:02713TZ	Dilation of Coronary Artery, Two Arteries with Radioactive Intraluminal Device, Percutaneous Approach
procedure	UMLS:ICD10PCS:02713Z6	Dilation of Coronary Artery, Two Arteries, Bifurcation, Percutaneous Approach
procedure	UMLS:ICD10PCS:02713ZZ	Dilation of Coronary Artery, Two Arteries, Percutaneous Approach
procedure	UMLS:ICD10PCS:0271446	Dilation of Coronary Artery, Two Arteries, Bifurcation, with Drug-eluting Intraluminal Device, Percutaneous Endoscopic Approach
procedure	UMLS:ICD10PCS:027144Z	Dilation of Coronary Artery, Two Arteries with Drug-eluting Intraluminal Device, Percutaneous Endoscopic Approach
procedure	UMLS:ICD10PCS:0271456	Dilation of Coronary Artery, Two Arteries, Bifurcation, with Two Drug-eluting Intraluminal Devices, Percutaneous Endoscopic Approach
procedure	UMLS:ICD10PCS:027145Z	Dilation of Coronary Artery, Two Arteries with Two Drug-eluting Intraluminal Devices, Percutaneous Endoscopic Approach
procedure	UMLS:ICD10PCS:0271466	Dilation of Coronary Artery, Two Arteries, Bifurcation, with Three Drug-eluting Intraluminal Devices, Percutaneous Endoscopic Approach

procedure	UMLS:ICD10PCS:027146Z	Dilation of Coronary Artery, Two Arteries with Three Drug-eluting Intraluminal Devices, Percutaneous Endoscopic Approach
procedure	UMLS:ICD10PCS:027147Z	Dilation of Coronary Artery, Two Arteries with Four or More Drug-eluting Intraluminal Devices, Percutaneous Endoscopic Approach
procedure	UMLS:ICD10PCS:02714D6	Dilation of Coronary Artery, Two Arteries, Bifurcation, with Intraluminal Device, Percutaneous Endoscopic Approach
procedure	UMLS:ICD10PCS:02714DZ	Dilation of Coronary Artery, Two Arteries with Intraluminal Device, Percutaneous Endoscopic Approach
procedure	UMLS:ICD10PCS:02714EZ	Dilation of Coronary Artery, Two Arteries with Two Intraluminal Devices, Percutaneous Endoscopic Approach
procedure	UMLS:ICD10PCS:02714FZ	Dilation of Coronary Artery, Two Arteries with Three Intraluminal Devices, Percutaneous Endoscopic Approach
procedure	UMLS:ICD10PCS:02714G6	Dilation of Coronary Artery, Two Arteries, Bifurcation, with Four or More Intraluminal Devices, Percutaneous Endoscopic Approach
procedure	UMLS:ICD10PCS:02714Z6	Dilation of Coronary Artery, Two Arteries, Bifurcation, Percutaneous Endoscopic Approach
procedure	UMLS:ICD10PCS:02714ZZ	Dilation of Coronary Artery, Two Arteries, Percutaneous Endoscopic Approach
procedure	UMLS:ICD10PCS:0272346	Dilation of Coronary Artery, Three Arteries, Bifurcation, with Drug-eluting Intraluminal Device, Percutaneous Approach
procedure	UMLS:ICD10PCS:027234Z	Dilation of Coronary Artery, Three Arteries with Drug-eluting Intraluminal Device, Percutaneous Approach
procedure	UMLS:ICD10PCS:0272356	Dilation of Coronary Artery, Three Arteries, Bifurcation, with Two Drug-eluting Intraluminal Devices, Percutaneous Approach
procedure	UMLS:ICD10PCS:027235Z	Dilation of Coronary Artery, Three Arteries with Two Drug-eluting Intraluminal Devices, Percutaneous Approach
procedure	UMLS:ICD10PCS:0272366	Dilation of Coronary Artery, Three Arteries, Bifurcation, with Three Drug-eluting Intraluminal Devices, Percutaneous Approach
procedure	UMLS:ICD10PCS:027236Z	Dilation of Coronary Artery, Three Arteries with Three Drug-eluting Intraluminal Devices, Percutaneous Approach
procedure	UMLS:ICD10PCS:0272376	Dilation of Coronary Artery, Three Arteries, Bifurcation, with Four or More Drug-eluting Intraluminal Devices, Percutaneous Approach

procedure	UMLS:ICD10PCS:027237Z	Dilation of Coronary Artery, Three Arteries with Four or More Drug-eluting Intraluminal Devices, Percutaneous Approach
procedure	UMLS:ICD10PCS:02723D6	Dilation of Coronary Artery, Three Arteries, Bifurcation, with Intraluminal Device, Percutaneous Approach
procedure	UMLS:ICD10PCS:02723DZ	Dilation of Coronary Artery, Three Arteries with Intraluminal Device, Percutaneous Approach
procedure	UMLS:ICD10PCS:02723E6	Dilation of Coronary Artery, Three Arteries, Bifurcation, with Two Intraluminal Devices, Percutaneous Approach
procedure	UMLS:ICD10PCS:02723EZ	Dilation of Coronary Artery, Three Arteries with Two Intraluminal Devices, Percutaneous Approach
procedure	UMLS:ICD10PCS:02723F6	Dilation of Coronary Artery, Three Arteries, Bifurcation, with Three Intraluminal Devices, Percutaneous Approach
procedure	UMLS:ICD10PCS:02723FZ	Dilation of Coronary Artery, Three Arteries with Three Intraluminal Devices, Percutaneous Approach
procedure	UMLS:ICD10PCS:02723G6	Dilation of Coronary Artery, Three Arteries, Bifurcation, with Four or More Intraluminal Devices, Percutaneous Approach
procedure	UMLS:ICD10PCS:02723GZ	Dilation of Coronary Artery, Three Arteries with Four or More Intraluminal Devices, Percutaneous Approach
procedure	UMLS:ICD10PCS:02723Z6	Dilation of Coronary Artery, Three Arteries, Bifurcation, Percutaneous Approach
procedure	UMLS:ICD10PCS:02723ZZ	Dilation of Coronary Artery, Three Arteries, Percutaneous Approach
procedure	UMLS:ICD10PCS:027244Z	Dilation of Coronary Artery, Three Arteries with Drug-eluting Intraluminal Device, Percutaneous Endoscopic Approach
procedure	UMLS:ICD10PCS:027245Z	Dilation of Coronary Artery, Three Arteries with Two Drug-eluting Intraluminal Devices, Percutaneous Endoscopic Approach
procedure	UMLS:ICD10PCS:027246Z	Dilation of Coronary Artery, Three Arteries with Three Drug-eluting Intraluminal Devices, Percutaneous Endoscopic Approach
procedure	UMLS:ICD10PCS:0272466	Dilation of Coronary Artery, Three Arteries, Bifurcation, with Three Drug-eluting Intraluminal Devices, Percutaneous Endoscopic Approach
procedure	UMLS:ICD10PCS:027247Z	Dilation of Coronary Artery, Three Arteries with Four or More Drug-eluting Intraluminal Devices, Percutaneous Endoscopic Approach
procedure	UMLS:ICD10PCS:02724DZ	Dilation of Coronary Artery, Three Arteries with Intraluminal Device, Percutaneous Endoscopic Approach

procedure	UMLS:ICD10PCS:02724ZZ	Dilation of Coronary Artery, Three Arteries, Percutaneous Endoscopic Approach
procedure	UMLS:ICD10PCS:0273346	Dilation of Coronary Artery, Four or More Arteries, Bifurcation, with Drug-eluting Intraluminal Device, Percutaneous Approach
procedure	UMLS:ICD10PCS:027334Z	Dilation of Coronary Artery, Four or More Arteries with Drug-eluting Intraluminal Device, Percutaneous Approach
procedure	UMLS:ICD10PCS:0273356	Dilation of Coronary Artery, Four or More Arteries, Bifurcation, with Two Drug-eluting Intraluminal Devices, Percutaneous Approach
procedure	UMLS:ICD10PCS:027335Z	Dilation of Coronary Artery, Four or More Arteries with Two Drug-eluting Intraluminal Devices, Percutaneous Approach
procedure	UMLS:ICD10PCS:0273366	Dilation of Coronary Artery, Four or More Arteries, Bifurcation, with Three Drug-eluting Intraluminal Devices, Percutaneous Approach
procedure	UMLS:ICD10PCS:027336Z	Dilation of Coronary Artery, Four or More Arteries with Three Drug-eluting Intraluminal Devices, Percutaneous Approach
procedure	UMLS:ICD10PCS:0273376	Dilation of Coronary Artery, Four or More Arteries, Bifurcation, with Four or More Drug-eluting Intraluminal Devices, Percutaneous Approach
procedure	UMLS:ICD10PCS:027337Z	Dilation of Coronary Artery, Four or More Arteries with Four or More Drug-eluting Intraluminal Devices, Percutaneous Approach
procedure	UMLS:ICD10PCS:02733D6	Dilation of Coronary Artery, Four or More Arteries, Bifurcation, with Intraluminal Device, Percutaneous Approach
procedure	UMLS:ICD10PCS:02733DZ	Dilation of Coronary Artery, Four or More Arteries with Intraluminal Device, Percutaneous Approach
procedure	UMLS:ICD10PCS:02733FZ	Dilation of Coronary Artery, Four or More Arteries with Three Intraluminal Devices, Percutaneous Approach
procedure	UMLS:ICD10PCS:02733G6	Dilation of Coronary Artery, Four or More Arteries, Bifurcation, with Four or More Intraluminal Devices, Percutaneous Approach
procedure	UMLS:ICD10PCS:02733GZ	Dilation of Coronary Artery, Four or More Arteries with Four or More Intraluminal Devices, Percutaneous Approach
procedure	UMLS:ICD10PCS:02733Z6	Dilation of Coronary Artery, Four or More Arteries, Bifurcation, Percutaneous Approach
procedure	UMLS:ICD10PCS:02733ZZ	Dilation of Coronary Artery, Four or More Arteries, Percutaneous Approach
procedure	UMLS:ICD10PCS:027344Z	Dilation of Coronary Artery, Four or More Arteries with Drug-eluting

		Intraluminal Device, Percutaneous Endoscopic Approach
procedure	UMLS:ICD10PCS:02734 ZZ	Dilation of Coronary Artery, Four or More Arteries, Percutaneous Endoscopic Approach
procedure	UMLS:ICD10PCS:02C03 Z6	Extirpation of Matter from Coronary Artery, One Artery, Bifurcation, Percutaneous Approach
procedure	UMLS:ICD10PCS:02C03 Z7	Extirpation of Matter from Coronary Artery, One Artery, Orbital Atherectomy Technique, Percutaneous Approach
procedure	UMLS:ICD10PCS:02C03 ZZ	Extirpation of Matter from Coronary Artery, One Artery, Percutaneous Approach
procedure	UMLS:ICD10PCS:02C04 Z6	Extirpation of Matter from Coronary Artery, One Artery, Bifurcation, Percutaneous Endoscopic Approach
procedure	UMLS:ICD10PCS:02C04 ZZ	Extirpation of Matter from Coronary Artery, One Artery, Percutaneous Endoscopic Approach
procedure	UMLS:ICD10PCS:02C13 Z6	Extirpation of Matter from Coronary Artery, Two Arteries, Bifurcation, Percutaneous Approach
procedure	UMLS:ICD10PCS:02C13 Z7	Extirpation of Matter from Coronary Artery, Two Arteries, Orbital Atherectomy Technique, Percutaneous Approach
procedure	UMLS:ICD10PCS:02C13 ZZ	Extirpation of Matter from Coronary Artery, Two Arteries, Percutaneous Approach
procedure	UMLS:ICD10PCS:02C14 Z6	Extirpation of Matter from Coronary Artery, Two Arteries, Bifurcation, Percutaneous Endoscopic Approach
procedure	UMLS:ICD10PCS:02C14 ZZ	Extirpation of Matter from Coronary Artery, Two Arteries, Percutaneous Endoscopic Approach
procedure	UMLS:ICD10PCS:02C23 Z6	Extirpation of Matter from Coronary Artery, Three Arteries, Bifurcation, Percutaneous Approach
procedure	UMLS:ICD10PCS:02C23 Z7	Extirpation of Matter from Coronary Artery, Three Arteries, Orbital Atherectomy Technique, Percutaneous Approach
procedure	UMLS:ICD10PCS:02C23 ZZ	Extirpation of Matter from Coronary Artery, Three Arteries, Percutaneous Approach
procedure	UMLS:ICD10PCS:02C24 Z6	Extirpation of Matter from Coronary Artery, Three Arteries, Bifurcation, Percutaneous Endoscopic Approach
procedure	UMLS:ICD10PCS:02C24 ZZ	Extirpation of Matter from Coronary Artery, Three Arteries, Percutaneous Endoscopic Approach
procedure	UMLS:ICD10PCS:02C33 Z6	Extirpation of Matter from Coronary Artery, Four or More Arteries, Bifurcation, Percutaneous Approach
procedure	UMLS:ICD10PCS:02C33 ZZ	Extirpation of Matter from Coronary Artery, Four or More Arteries, Percutaneous Approach

		procedure	UMLS:ICD10PCS:02C34ZZ	Extirpation of Matter from Coronary Artery, Four or More Arteries, Percutaneous Endoscopic Approach
**Group 3**				
**Group 3A AMI**				
must have		diagnosis	UMLS:ICD10CM:I21	Acute myocardial infarction
date constraint		The terms in this group occurred at any time		
event relationship		Any instance of labs occurred on the same date as the first instance of AMI		
**Group 3B labs**				
must have		laboratory	TNX:9008	Erythrocyte distribution width [Ratio] (at least 13.50 %)

Supplementary Table S2. Query Criteria for Cohort 2 (AMI cases with RDW < 13.5%)

This query was run on the network US Collaborative Network with 70 HCO(s) queried and 70 HCO(s) responded. A total of 48 provider(s) responded with patients. The final cohort included 43,714 patients who matched the query criteria listed in the table below.

**Ungrouped terms**				
must have		demographics	Age	Age (at least 18 years (most recent occurrence))
	and any of	demographics	UMLS:HL7V3.0:Gender: M	Male
		demographics	UMLS:HL7V3.0:Gender: F	Female
**Group 1**				
**AMI**				
must have		diagnosis	UMLS:ICD10CM:I21	Acute myocardial infarction
date constraint		The terms in this group occurred between Jan 1, 2019 and Dec 31, 2023		
**Group 2**				
**Group 2A AMI**				
must have		diagnosis	UMLS:ICD10CM:I21	Acute myocardial infarction
date constraint		The terms in this group occurred at any time		
event relationship		Any instance of PCI occurred within 3 days before or up to 3 days after the first instance of AMI		
**Group 2B PCI**				
must have	any of	procedure	UMLS:ICD10PCS:02F0	Heart And Great Vessels / Fragmentation / Coronary Artery, One Artery
		procedure	UMLS:ICD10PCS:02F1	Heart And Great Vessels / Fragmentation / Coronary Artery, Two Arteries
		procedure	UMLS:ICD10PCS:02F2	Heart And Great Vessels / Fragmentation / Coronary Artery, Three Arteries
		procedure	UMLS:ICD10PCS:02F3	Heart And Great Vessels / Fragmentation / Coronary Artery, Four Or More Arteries
		procedure	UMLS:ICD10PCS:0270346	Dilation of Coronary Artery, One Artery, Bifurcation, with Drug-eluting Intraluminal Device, Percutaneous Approach
		procedure	UMLS:ICD10PCS:027034Z	Dilation of Coronary Artery, One Artery with Drug-eluting Intraluminal Device, Percutaneous Approach
		procedure	UMLS:ICD10PCS:0270356	Dilation of Coronary Artery, One Artery, Bifurcation, with Two Drug-eluting Intraluminal Devices, Percutaneous Approach
		procedure	UMLS:ICD10PCS:027035Z	Dilation of Coronary Artery, One Artery with Two Drug-eluting Intraluminal Devices, Percutaneous Approach
		procedure	UMLS:ICD10PCS:0270366	Dilation of Coronary Artery, One Artery, Bifurcation, with Three Drug-eluting Intraluminal Devices, Percutaneous Approach
		procedure	UMLS:ICD10PCS:027036Z	Dilation of Coronary Artery, One Artery with Three Drug-eluting Intraluminal Devices, Percutaneous Approach

procedure	UMLS:ICD10PCS:0270376	Dilation of Coronary Artery, One Artery, Bifurcation, with Four or More Drug-eluting Intraluminal Devices, Percutaneous Approach
procedure	UMLS:ICD10PCS:027037Z	Dilation of Coronary Artery, One Artery with Four or More Drug-eluting Intraluminal Devices, Percutaneous Approach
procedure	UMLS:ICD10PCS:02703D6	Dilation of Coronary Artery, One Artery, Bifurcation, with Intraluminal Device, Percutaneous Approach
procedure	UMLS:ICD10PCS:02703E6	Dilation of Coronary Artery, One Artery, Bifurcation, with Two Intraluminal Devices, Percutaneous Approach
procedure	UMLS:ICD10PCS:02703DZ	Dilation of Coronary Artery, One Artery with Intraluminal Device, Percutaneous Approach
procedure	UMLS:ICD10PCS:02703EZ	Dilation of Coronary Artery, One Artery with Two Intraluminal Devices, Percutaneous Approach
procedure	UMLS:ICD10PCS:02703F6	Dilation of Coronary Artery, One Artery, Bifurcation, with Three Intraluminal Devices, Percutaneous Approach
procedure	UMLS:ICD10PCS:02703FZ	Dilation of Coronary Artery, One Artery with Three Intraluminal Devices, Percutaneous Approach
procedure	UMLS:ICD10PCS:02703G6	Dilation of Coronary Artery, One Artery, Bifurcation, with Four or More Intraluminal Devices, Percutaneous Approach
procedure	UMLS:ICD10PCS:02703GZ	Dilation of Coronary Artery, One Artery with Four or More Intraluminal Devices, Percutaneous Approach
procedure	UMLS:ICD10PCS:02703T6	Dilation of Coronary Artery, One Artery, Bifurcation, with Radioactive Intraluminal Device, Percutaneous Approach
procedure	UMLS:ICD10PCS:02703TZ	Dilation of Coronary Artery, One Artery with Radioactive Intraluminal Device, Percutaneous Approach
procedure	UMLS:ICD10PCS:02703Z6	Dilation of Coronary Artery, One Artery, Bifurcation, Percutaneous Approach
procedure	UMLS:ICD10PCS:02703ZZ	Dilation of Coronary Artery, One Artery, Percutaneous Approach
procedure	UMLS:ICD10PCS:0270446	Dilation of Coronary Artery, One Artery, Bifurcation, with Drug-eluting Intraluminal Device, Percutaneous Endoscopic Approach
procedure	UMLS:ICD10PCS:027044Z	Dilation of Coronary Artery, One Artery with Drug-eluting Intraluminal Device, Percutaneous Endoscopic Approach
procedure	UMLS:ICD10PCS:0270456	Dilation of Coronary Artery, One Artery, Bifurcation, with Two Drug-eluting Intraluminal Devices, Percutaneous Endoscopic Approach
procedure	UMLS:ICD10PCS:027045Z	Dilation of Coronary Artery, One Artery with Two Drug-eluting Intraluminal

		Devices, Percutaneous Endoscopic Approach
procedure	UMLS:ICD10PCS:027046Z	Dilation of Coronary Artery, One Artery with Three Drug-eluting Intraluminal Devices, Percutaneous Endoscopic Approach
procedure	UMLS:ICD10PCS:027047Z	Dilation of Coronary Artery, One Artery with Four or More Drug-eluting Intraluminal Devices, Percutaneous Endoscopic Approach
procedure	UMLS:ICD10PCS:02704D6	Dilation of Coronary Artery, One Artery, Bifurcation, with Intraluminal Device, Percutaneous Endoscopic Approach
procedure	UMLS:ICD10PCS:02704DZ	Dilation of Coronary Artery, One Artery with Intraluminal Device, Percutaneous Endoscopic Approach
procedure	UMLS:ICD10PCS:02704EZ	Dilation of Coronary Artery, One Artery with Two Intraluminal Devices, Percutaneous Endoscopic Approach
procedure	UMLS:ICD10PCS:02704FZ	Dilation of Coronary Artery, One Artery with Three Intraluminal Devices, Percutaneous Endoscopic Approach
procedure	UMLS:ICD10PCS:02704Z6	Dilation of Coronary Artery, One Artery, Bifurcation, Percutaneous Endoscopic Approach
procedure	UMLS:ICD10PCS:02704ZZ	Dilation of Coronary Artery, One Artery, Percutaneous Endoscopic Approach
procedure	UMLS:ICD10PCS:0271346	Dilation of Coronary Artery, Two Arteries, Bifurcation, with Drug-eluting Intraluminal Device, Percutaneous Approach
procedure	UMLS:ICD10PCS:027134Z	Dilation of Coronary Artery, Two Arteries with Drug-eluting Intraluminal Device, Percutaneous Approach
procedure	UMLS:ICD10PCS:0271356	Dilation of Coronary Artery, Two Arteries, Bifurcation, with Two Drug-eluting Intraluminal Devices, Percutaneous Approach
procedure	UMLS:ICD10PCS:027135Z	Dilation of Coronary Artery, Two Arteries with Two Drug-eluting Intraluminal Devices, Percutaneous Approach
procedure	UMLS:ICD10PCS:0271366	Dilation of Coronary Artery, Two Arteries, Bifurcation, with Three Drug-eluting Intraluminal Devices, Percutaneous Approach
procedure	UMLS:ICD10PCS:027136Z	Dilation of Coronary Artery, Two Arteries with Three Drug-eluting Intraluminal Devices, Percutaneous Approach
procedure	UMLS:ICD10PCS:0271376	Dilation of Coronary Artery, Two Arteries, Bifurcation, with Four or More Drug-eluting Intraluminal Devices, Percutaneous Approach
procedure	UMLS:ICD10PCS:027137Z	Dilation of Coronary Artery, Two Arteries with Four or More Drug-eluting Intraluminal Devices, Percutaneous Approach

procedure	UMLS:ICD10PCS:02713D6	Dilation of Coronary Artery, Two Arteries, Bifurcation, with Intraluminal Device, Percutaneous Approach
procedure	UMLS:ICD10PCS:02713DZ	Dilation of Coronary Artery, Two Arteries with Intraluminal Device, Percutaneous Approach
procedure	UMLS:ICD10PCS:02713E6	Dilation of Coronary Artery, Two Arteries, Bifurcation, with Two Intraluminal Devices, Percutaneous Approach
procedure	UMLS:ICD10PCS:02713EZ	Dilation of Coronary Artery, Two Arteries with Two Intraluminal Devices, Percutaneous Approach
procedure	UMLS:ICD10PCS:02713F6	Dilation of Coronary Artery, Two Arteries, Bifurcation, with Three Intraluminal Devices, Percutaneous Approach
procedure	UMLS:ICD10PCS:02713FZ	Dilation of Coronary Artery, Two Arteries with Three Intraluminal Devices, Percutaneous Approach
procedure	UMLS:ICD10PCS:02713G6	Dilation of Coronary Artery, Two Arteries, Bifurcation, with Four or More Intraluminal Devices, Percutaneous Approach
procedure	UMLS:ICD10PCS:02713GZ	Dilation of Coronary Artery, Two Arteries with Four or More Intraluminal Devices, Percutaneous Approach
procedure	UMLS:ICD10PCS:02713T6	Dilation of Coronary Artery, Two Arteries, Bifurcation, with Radioactive Intraluminal Device, Percutaneous Approach
procedure	UMLS:ICD10PCS:02713TZ	Dilation of Coronary Artery, Two Arteries with Radioactive Intraluminal Device, Percutaneous Approach
procedure	UMLS:ICD10PCS:02713Z6	Dilation of Coronary Artery, Two Arteries, Bifurcation, Percutaneous Approach
procedure	UMLS:ICD10PCS:02713ZZ	Dilation of Coronary Artery, Two Arteries, Percutaneous Approach
procedure	UMLS:ICD10PCS:0271446	Dilation of Coronary Artery, Two Arteries, Bifurcation, with Drug-eluting Intraluminal Device, Percutaneous Endoscopic Approach
procedure	UMLS:ICD10PCS:027144Z	Dilation of Coronary Artery, Two Arteries with Drug-eluting Intraluminal Device, Percutaneous Endoscopic Approach
procedure	UMLS:ICD10PCS:0271456	Dilation of Coronary Artery, Two Arteries, Bifurcation, with Two Drug-eluting Intraluminal Devices, Percutaneous Endoscopic Approach
procedure	UMLS:ICD10PCS:027145Z	Dilation of Coronary Artery, Two Arteries with Two Drug-eluting Intraluminal Devices, Percutaneous Endoscopic Approach
procedure	UMLS:ICD10PCS:0271466	Dilation of Coronary Artery, Two Arteries, Bifurcation, with Three Drug-eluting Intraluminal Devices, Percutaneous Endoscopic Approach

procedure	UMLS:ICD10PCS:027146Z	Dilation of Coronary Artery, Two Arteries with Three Drug-eluting Intraluminal Devices, Percutaneous Endoscopic Approach
procedure	UMLS:ICD10PCS:027147Z	Dilation of Coronary Artery, Two Arteries with Four or More Drug-eluting Intraluminal Devices, Percutaneous Endoscopic Approach
procedure	UMLS:ICD10PCS:02714D6	Dilation of Coronary Artery, Two Arteries, Bifurcation, with Intraluminal Device, Percutaneous Endoscopic Approach
procedure	UMLS:ICD10PCS:02714DZ	Dilation of Coronary Artery, Two Arteries with Intraluminal Device, Percutaneous Endoscopic Approach
procedure	UMLS:ICD10PCS:02714EZ	Dilation of Coronary Artery, Two Arteries with Two Intraluminal Devices, Percutaneous Endoscopic Approach
procedure	UMLS:ICD10PCS:02714FZ	Dilation of Coronary Artery, Two Arteries with Three Intraluminal Devices, Percutaneous Endoscopic Approach
procedure	UMLS:ICD10PCS:02714G6	Dilation of Coronary Artery, Two Arteries, Bifurcation, with Four or More Intraluminal Devices, Percutaneous Endoscopic Approach
procedure	UMLS:ICD10PCS:02714Z6	Dilation of Coronary Artery, Two Arteries, Bifurcation, Percutaneous Endoscopic Approach
procedure	UMLS:ICD10PCS:02714ZZ	Dilation of Coronary Artery, Two Arteries, Percutaneous Endoscopic Approach
procedure	UMLS:ICD10PCS:0272346	Dilation of Coronary Artery, Three Arteries, Bifurcation, with Drug-eluting Intraluminal Device, Percutaneous Approach
procedure	UMLS:ICD10PCS:027234Z	Dilation of Coronary Artery, Three Arteries with Drug-eluting Intraluminal Device, Percutaneous Approach
procedure	UMLS:ICD10PCS:0272356	Dilation of Coronary Artery, Three Arteries, Bifurcation, with Two Drug-eluting Intraluminal Devices, Percutaneous Approach
procedure	UMLS:ICD10PCS:027235Z	Dilation of Coronary Artery, Three Arteries with Two Drug-eluting Intraluminal Devices, Percutaneous Approach
procedure	UMLS:ICD10PCS:0272366	Dilation of Coronary Artery, Three Arteries, Bifurcation, with Three Drug-eluting Intraluminal Devices, Percutaneous Approach
procedure	UMLS:ICD10PCS:027236Z	Dilation of Coronary Artery, Three Arteries with Three Drug-eluting Intraluminal Devices, Percutaneous Approach
procedure	UMLS:ICD10PCS:0272376	Dilation of Coronary Artery, Three Arteries, Bifurcation, with Four or More Drug-eluting Intraluminal Devices, Percutaneous Approach

procedure	UMLS:ICD10PCS:027237Z	Dilation of Coronary Artery, Three Arteries with Four or More Drug-eluting Intraluminal Devices, Percutaneous Approach
procedure	UMLS:ICD10PCS:02723D6	Dilation of Coronary Artery, Three Arteries, Bifurcation, with Intraluminal Device, Percutaneous Approach
procedure	UMLS:ICD10PCS:02723DZ	Dilation of Coronary Artery, Three Arteries with Intraluminal Device, Percutaneous Approach
procedure	UMLS:ICD10PCS:02723E6	Dilation of Coronary Artery, Three Arteries, Bifurcation, with Two Intraluminal Devices, Percutaneous Approach
procedure	UMLS:ICD10PCS:02723EZ	Dilation of Coronary Artery, Three Arteries with Two Intraluminal Devices, Percutaneous Approach
procedure	UMLS:ICD10PCS:02723F6	Dilation of Coronary Artery, Three Arteries, Bifurcation, with Three Intraluminal Devices, Percutaneous Approach
procedure	UMLS:ICD10PCS:02723FZ	Dilation of Coronary Artery, Three Arteries with Three Intraluminal Devices, Percutaneous Approach
procedure	UMLS:ICD10PCS:02723G6	Dilation of Coronary Artery, Three Arteries, Bifurcation, with Four or More Intraluminal Devices, Percutaneous Approach
procedure	UMLS:ICD10PCS:02723GZ	Dilation of Coronary Artery, Three Arteries with Four or More Intraluminal Devices, Percutaneous Approach
procedure	UMLS:ICD10PCS:02723Z6	Dilation of Coronary Artery, Three Arteries, Bifurcation, Percutaneous Approach
procedure	UMLS:ICD10PCS:02723ZZ	Dilation of Coronary Artery, Three Arteries, Percutaneous Approach
procedure	UMLS:ICD10PCS:027244Z	Dilation of Coronary Artery, Three Arteries with Drug-eluting Intraluminal Device, Percutaneous Endoscopic Approach
procedure	UMLS:ICD10PCS:027245Z	Dilation of Coronary Artery, Three Arteries with Two Drug-eluting Intraluminal Devices, Percutaneous Endoscopic Approach
procedure	UMLS:ICD10PCS:027246Z	Dilation of Coronary Artery, Three Arteries with Three Drug-eluting Intraluminal Devices, Percutaneous Endoscopic Approach
procedure	UMLS:ICD10PCS:0272466	Dilation of Coronary Artery, Three Arteries, Bifurcation, with Three Drug-eluting Intraluminal Devices, Percutaneous Endoscopic Approach
procedure	UMLS:ICD10PCS:027247Z	Dilation of Coronary Artery, Three Arteries with Four or More Drug-eluting Intraluminal Devices, Percutaneous Endoscopic Approach
procedure	UMLS:ICD10PCS:02724DZ	Dilation of Coronary Artery, Three Arteries with Intraluminal Device, Percutaneous Endoscopic Approach

procedure	UMLS:ICD10PCS:02724ZZ	Dilation of Coronary Artery, Three Arteries, Percutaneous Endoscopic Approach
procedure	UMLS:ICD10PCS:0273346	Dilation of Coronary Artery, Four or More Arteries, Bifurcation, with Drug-eluting Intraluminal Device, Percutaneous Approach
procedure	UMLS:ICD10PCS:027334Z	Dilation of Coronary Artery, Four or More Arteries with Drug-eluting Intraluminal Device, Percutaneous Approach
procedure	UMLS:ICD10PCS:0273356	Dilation of Coronary Artery, Four or More Arteries, Bifurcation, with Two Drug-eluting Intraluminal Devices, Percutaneous Approach
procedure	UMLS:ICD10PCS:027335Z	Dilation of Coronary Artery, Four or More Arteries with Two Drug-eluting Intraluminal Devices, Percutaneous Approach
procedure	UMLS:ICD10PCS:0273366	Dilation of Coronary Artery, Four or More Arteries, Bifurcation, with Three Drug-eluting Intraluminal Devices, Percutaneous Approach
procedure	UMLS:ICD10PCS:027336Z	Dilation of Coronary Artery, Four or More Arteries with Three Drug-eluting Intraluminal Devices, Percutaneous Approach
procedure	UMLS:ICD10PCS:0273376	Dilation of Coronary Artery, Four or More Arteries, Bifurcation, with Four or More Drug-eluting Intraluminal Devices, Percutaneous Approach
procedure	UMLS:ICD10PCS:027337Z	Dilation of Coronary Artery, Four or More Arteries with Four or More Drug-eluting Intraluminal Devices, Percutaneous Approach
procedure	UMLS:ICD10PCS:02733D6	Dilation of Coronary Artery, Four or More Arteries, Bifurcation, with Intraluminal Device, Percutaneous Approach
procedure	UMLS:ICD10PCS:02733DZ	Dilation of Coronary Artery, Four or More Arteries with Intraluminal Device, Percutaneous Approach
procedure	UMLS:ICD10PCS:02733FZ	Dilation of Coronary Artery, Four or More Arteries with Three Intraluminal Devices, Percutaneous Approach
procedure	UMLS:ICD10PCS:02733G6	Dilation of Coronary Artery, Four or More Arteries, Bifurcation, with Four or More Intraluminal Devices, Percutaneous Approach
procedure	UMLS:ICD10PCS:02733GZ	Dilation of Coronary Artery, Four or More Arteries with Four or More Intraluminal Devices, Percutaneous Approach
procedure	UMLS:ICD10PCS:02733Z6	Dilation of Coronary Artery, Four or More Arteries, Bifurcation, Percutaneous Approach
procedure	UMLS:ICD10PCS:02733ZZ	Dilation of Coronary Artery, Four or More Arteries, Percutaneous Approach
procedure	UMLS:ICD10PCS:027344Z	Dilation of Coronary Artery, Four or More Arteries with Drug-eluting

		Intraluminal Device, Percutaneous Endoscopic Approach
procedure	UMLS:ICD10PCS:02734 ZZ	Dilation of Coronary Artery, Four or More Arteries, Percutaneous Endoscopic Approach
procedure	UMLS:ICD10PCS:02C03 Z6	Extirpation of Matter from Coronary Artery, One Artery, Bifurcation, Percutaneous Approach
procedure	UMLS:ICD10PCS:02C03 Z7	Extirpation of Matter from Coronary Artery, One Artery, Orbital Atherectomy Technique, Percutaneous Approach
procedure	UMLS:ICD10PCS:02C03 ZZ	Extirpation of Matter from Coronary Artery, One Artery, Percutaneous Approach
procedure	UMLS:ICD10PCS:02C04 Z6	Extirpation of Matter from Coronary Artery, One Artery, Bifurcation, Percutaneous Endoscopic Approach
procedure	UMLS:ICD10PCS:02C04 ZZ	Extirpation of Matter from Coronary Artery, One Artery, Percutaneous Endoscopic Approach
procedure	UMLS:ICD10PCS:02C13 Z6	Extirpation of Matter from Coronary Artery, Two Arteries, Bifurcation, Percutaneous Approach
procedure	UMLS:ICD10PCS:02C13 Z7	Extirpation of Matter from Coronary Artery, Two Arteries, Orbital Atherectomy Technique, Percutaneous Approach
procedure	UMLS:ICD10PCS:02C13 ZZ	Extirpation of Matter from Coronary Artery, Two Arteries, Percutaneous Approach
procedure	UMLS:ICD10PCS:02C14 Z6	Extirpation of Matter from Coronary Artery, Two Arteries, Bifurcation, Percutaneous Endoscopic Approach
procedure	UMLS:ICD10PCS:02C14 ZZ	Extirpation of Matter from Coronary Artery, Two Arteries, Percutaneous Endoscopic Approach
procedure	UMLS:ICD10PCS:02C23 Z6	Extirpation of Matter from Coronary Artery, Three Arteries, Bifurcation, Percutaneous Approach
procedure	UMLS:ICD10PCS:02C23 Z7	Extirpation of Matter from Coronary Artery, Three Arteries, Orbital Atherectomy Technique, Percutaneous Approach
procedure	UMLS:ICD10PCS:02C23 ZZ	Extirpation of Matter from Coronary Artery, Three Arteries, Percutaneous Approach
procedure	UMLS:ICD10PCS:02C24 Z6	Extirpation of Matter from Coronary Artery, Three Arteries, Bifurcation, Percutaneous Endoscopic Approach
procedure	UMLS:ICD10PCS:02C24 ZZ	Extirpation of Matter from Coronary Artery, Three Arteries, Percutaneous Endoscopic Approach
procedure	UMLS:ICD10PCS:02C33 Z6	Extirpation of Matter from Coronary Artery, Four or More Arteries, Bifurcation, Percutaneous Approach
procedure	UMLS:ICD10PCS:02C33 ZZ	Extirpation of Matter from Coronary Artery, Four or More Arteries, Percutaneous Approach

		procedure	UMLS:ICD10PCS:02C34ZZ	Extirpation of Matter from Coronary Artery, Four or More Arteries, Percutaneous Endoscopic Approach
**Group 3**				
**Group 3A AMI**				
must have		diagnosis	UMLS:ICD10CM:I21	Acute myocardial infarction
date constraint		The terms in this group occurred at any time		
event relationship		Any instance of labs occurred on the same date as the first instance of AMI		
**Group 3B labs**				
must have		laboratory	TNX:9008	Erythrocyte distribution width [Ratio] (at most 13.40 %)

Supplementary Table S3. ICD-10-CM code definition of comorbidities and outcomes.

Comorbidities	ICD-10-CM
Hypertensive diseases	I10-I15
Ischemic heart diseases	I20-I25
Atrioventricular and left bundle-branch block	I44
Other conduction disorders	I45
Heart failure	I50
Cerebrovascular diseases	I60-I69
Diabetes mellitus	E08-E13
Disorders of lipoprotein metabolism and other lipidemias	E78
Overweight, obesity and other hyperalimentation	E65-E68
Chronic lower respiratory diseases	J40-J4A
Chronic kidney disease	N18
Neoplasms	C00-D49
Gastro-esophageal reflux disease	K21
Chronic liver diseases	K70-K77
Syncope and collapse	R55
Nutritional anemias	D50-D53
Hemolytic anemias	D55-D59
Aplastic and other anemias and other bone marrow failure syndromes	D60-D64
Parkinson's disease	G20
Alzheimer's disease	G30
Epilepsy and recurrent seizures	G40
Vascular dementia	F01
Unspecified dementia	F03
Mental and behavioral disorders due to psychoactive substance use	F10-F19
Mood [affective] disorders	F30-F39
Schizophrenia, schizotypal, delusional, and other non-mood psychotic disorders	F20-F29
**Acute myocardial infarction type**	**ICD-10-CM**
ST-elevation myocardial infarction	I21.0-I21.3
Non-ST-elevation myocardial infarction	I21.4, I21.9
**Outcomes definition**	**ICD-10-CM**
Major adverse cardiovascular events	I21, I22, I60-63, I50, R57.0, I45, I47-I49
Heart failure	I50
Cardiogenic shock	R57.0
Acute myocardial infarction	I21, I22
Cerebrovascular accidents	I60-I63
Ventricular tachycardia and fibrillation	I47.2, I49.0
Cardiac arrhythmia	I45, I47-I49
